# RNase III-Binding-mRNAs Revealed Novel Complementary Transcripts in *Streptomyces*

**DOI:** 10.3389/fmicb.2017.02693

**Published:** 2018-01-15

**Authors:** Dita Šetinová, Klára Šmídová, Pavel Pohl, Inesa Musić, Jan Bobek

**Affiliations:** ^1^First Faculty of Medicine, Institute of Immunology and Microbiology, Charles University, Prague, Czechia; ^2^Chemistry Department, Faculty of Science, J. E. Purkinje University, Ústí nad Labem, Czechia; ^3^Institute of Microbiology, Academy of Sciences of the Czech Republic, Prague, Czechia

**Keywords:** *cis*-antisense RNA, RNase III, *Streptomyces*, antibiotics, gene expression control

## Abstract

*cis*-Antisense RNAs (asRNAs) provide very simple and effective gene expression control due to the perfect complementarity between regulated and regulatory transcripts. In *Streptomyces*, the antibiotic-producing clade, the antisense control system is not yet understood, although it might direct the organism's complex development. Initial studies in *Streptomyces* have found a number of asRNAs. Apart from this, hundreds of mRNAs have been shown to bind RNase III, the double strand-specific endoribonuclease. In this study, we tested 17 mRNAs that have been previously co-precipitated with RNase III for antisense expression. Our RACE mapping showed that all of these mRNAs possess cognate asRNA. Additional tests for antisense expression uncovered *as-adpA, as-rnc, as3983, as-sigB, as-sigH*, and *as-sigR* RNAs. Northern blots detected the expression profiles of 18 novel transcripts. Noteworthy, we also found that only a minority of asRNAs respond to the absence of RNase III enzyme by increasing their cellular levels. Our findings suggest that antisense expression is widespread in *Streptomyces*, including genes of such important developmental regulators, as AdpA, RNase III, and sigma factors.

## Introduction

Bacterial small RNAs are important post-transcriptional regulators that control a variety of cell processes. A large majority of these RNAs acts on target mRNAs via base pairing, an antisense mechanism that leads to positive or negative regulation of the target gene, as reviewed in Thomason (Thomason and Storz, 2010). Such antisense RNAs fall into two groups: *cis*- (asRNAs) and *trans*-encoded (sRNAs) (Romby and Charpentier, 2010).

The *trans*-acting sRNAs are encoded distinctly from their target mRNA(s), which is also the reason why the sRNA-mRNA pair mostly shares reduced complementarity. On the other hand, the sRNAs are usually able to act on multiple targets. The *trans*-encoded sRNAs have been extensively characterized and discussed in many reviews (Papenfort and Vogel, 2009; Waters and Storz, 2009). The limited base pairing requires the RNA chaperone protein Hfq in a number of bacteria. However, Hfq is either missing or its homolog has not yet been found in several bacterial clades, including Actinomycetes (Sun et al., 2002).

Reported proportions of *cis-*antisense expression in various bacteria vary from 13% in *Bacillus subtilis* (Nicolas et al., 2012), 27% in *Synechocystis* PCC6803 (Mitschke et al., 2011a), 30% in *Anabaena* (Mitschke et al., 2011b), 46% in *Helicobacter pylori* (Sharma et al., 2010), and up to 49% in *Staphylococcus aureus* (Lasa et al., 2011). These transcripts are encoded on the DNA strand opposite to their specific targets. Both asRNA and mRNA are produced by overlapping transcription, thus sharing perfect complementarity (summarized in Lasa et al., 2012). The ability of asRNAs to modulate mRNA levels is not only mediated by post-transcriptional mechanisms but they may also directly impact transcription due to collisions between RNA polymerases traveling in opposite directions [transcription interference, for review see Thomason (Thomason and Storz, 2010)]. The mechanisms of post-transcriptional action of asRNAs may be divided into two groups: (i) asRNA influences the stability of the target mRNA by either tagging for degradation or stabilizing its structure and/or (ii) asRNA affects translation either by blocking or promoting ribosome access to the ribosome binding site (Geissmann et al., 2009; Lasa et al., 2011).

In many cases of negative antisense control, the sense-antisense RNA complex formation results in its rapid cleavage. In many bacteria, two dominant endoribonucleases, RNase E and RNase III, are involved in the degradation process. The RNase E enzyme, as a member of the degradosome complex with Hfq in *E. coli*, cleaves single-stranded RNA (reviewed in Carpousis et al., 2009). The RNase III enzyme cleaves double-stranded RNA (MacRae and Doudna, 2007). Although RNase III was initially shown to be associated with the processing of ribosomal RNAs (Carpousis et al., 2009; Taverniti et al., 2011), its involvement in the degradation of sense/antisense pairs is being reported in an increasing number of publications (Blomberg et al., 1990; Gerdes et al., 1992; Lasa et al., 2011, 2012; Durand et al., 2012; Lioliou et al., 2012; Lybecker et al., 2014a,b; Le Rhun et al., 2016).

In *Staphylococcus aureus*, deep sequencing of the short RNA fraction revealed a massive accumulation of 22-nucleotide RNA fragments generated by the RNase III cleavage of paired transcripts (Lasa et al., 2011). More than 75% of the fragments corresponded to the overlapping transcription from most regions of the chromosome. The number of short RNA fragments was significantly decreased in an RNase III-deletion strain. In contrast, such a collection of short RNA fragments was not found when using a similar transcriptome analysis for the Gram-negative bacterium *Salmonella enterica* (Viegas et al., 2007).

Bacteria of the genus *Streptomyces* undergo a complex mycelial life cycle. Their growth starts with the germination of spores that develop into a vegetative mycelium of branching hyphae. Subsequent development of aerial hyphae is considered to be a cell response to nutrient depletion (Chater and Losick, 1997). At this stage part of the vegetative mycelium is lysed and can be used as a nutrient source, while the synthesis of antibiotics reaches its maximum presumably to avoid competitive organisms. Eventually, the aerial hyphae are dissected into spores by sporulation septa, producing chains of uninucleoid spores.

The complexity of the morphological and physiological differentiation in *Streptomyces* can be documented by the existence of more than 900 transcriptional protein regulators that control the metabolic and developmental transitions. Among them, over 60 sigma factors have been identified thus far (Gruber and Gross, 2003). Besides sigma factors, one of the most pleiotropic transcription regulators is AdpA. AdpA is expressed in an A-factor-dependent manner in *Streptomyces griseus* and acts as a transcriptional repressor as well as an activator thus controlling expression of several hundred genes during *Streptomyces* development (Higo et al., 2012). In *Streptomyces coelicolor*, not only could AdpA-mRNA bind purified RNase III *in vitro*, but, as also shown, AdpA and RNase III coordinated the expression of each other in a posttranscriptional feedback loop (Xu et al., 2010). This finding may rationally explain the *rnc* (RNase III-deficient) mutant phenotype that affects expression of genes involved in sporulation and antibiotic production.

Although RNase III from the *Streptomyces* genus was recently shown to assist processing of ribosomal RNAs (Jones et al., 2014), it came to light as a global regulator of antibiotic biosynthesis (Adamidis and Champness, 1992; Aceti and Champness, 1998; Huang et al., 2005; Gatewood et al., 2012; Lee et al., 2013; Jones et al., 2014). In *Streptomyces coelicolor*, the deletion of the gene encoding RNase III [*rnc* gene, also termed as *absB* (Adamidis and Champness, 1992)] leads to a severely reduced production of at least four antibiotics (actinorhodin, undecylprodigiosin, CDA, and methylenomycin) (Price et al., 1999; Chang et al., 2005; Sello and Buttner, 2008). Microarray analysis was used to compare the levels of gene expression in the *S. coelicolor* parental strain and the RNase III mutant strain (Huang et al., 2005). A wide effect of the ribonuclease was found, mainly on genes connected with sporulation and antibiotic production. In the *rnc* mutant strain, sporulation genes were up-regulated, whereas activators of the antibiotic biosynthetic pathways (e.g., *actII-ORF4, redD, redZ*, and *cdaR*) were down-regulated, which is consistent with defects in antibiotic production. However, Strakova (Strakova et al., 2013) revealed that *rnc* expression is activated from the first hour of germination, suggesting a more general role for RNase III, either in ribosomal RNA or in asRNA processing. Subsequent microarray and co-immunoprecipitation analyses, performed by Gatewood (Gatewood et al., 2012), revealed at least 777 mRNAs bound by the RNase III enzyme. The authors also showed that the absence of the enzyme directly or indirectly affected the levels of hundreds of mRNAs and at least two small RNAs. These very valuable results greatly inspired the work described in this paper.

Confirmation of the expected employment of small RNAs in the regulation of cell processes, including primary metabolism, developmental transitions, antibiotic production, and various stress responses is being increasingly reported (Palecková et al., 2007; Pánek et al., 2008; Swiercz et al., 2008; D'Alia et al., 2010; Mikulík et al., 2014). Hundreds of *cis-*acting asRNAs were identified using RNA sequencing in two recent studies (Vockenhuber et al., 2011; Moody et al., 2013). Here, we further exploited Gatewood's results to see if there are connections between asRNAs and RNase III, which have not yet been reported in *Streptomyces*. We selected 17 mRNAs that are bound by the RNase III enzyme *in vivo* as stated in Gatewood et al. (2012) to check if they possessed an antisense transcript. Surprisingly, the search for asRNAs within the selected group of mRNAs was 100% successful. Moreover, additional analyses revealed antisense transcripts to selected mRNAs that encode RNase III and several transcription regulators (AdpA, SigB, SigH, and SigR).

Although our data did not elucidate the exact role of newly found asRNAs in the RNase III-degradation pathway, the current findings further demonstrate that the antisense mechanism is widely present in *Streptomyces* and antisense RNAs are possibly involved in developmental and antibiotic synthesis control.

## Materials and methods

### Bacterial strain, cultivation

In this study, the *Streptomyces coelicolor* wild-type (wt) strain M145 (Kieser et al., 2000) and its RNase III-deletion strain derivative [*rnc*, M145 *rnc::aac*(3)IV (Sello and Buttner, 2008)] were used. 10^8^ spores were inoculated on solid R2YE medium (Kieser et al., 2000) covered by cellophane at 29°C. Bacterial samples were collected after 24, 48, and 72 h of cultivation, where each time point represented a different developmental stage, i.e., vegetative mycelium, aerial mycelium, and spores.

### RNA isolation

Total RNA was isolated using a TRIzol method (Van Dessel et al., 2004). Harvested cells were immediately submerged in TRIzol reagent (Ambion) on ice (1 ml of TRIzol per 50 cm^2^ of culture dish surface area). Five glass beads (3 mm in diameter) were added to the cell suspension. The cells were disrupted using a Minilys homogenizer (Precellys) twice for 2 min at 3,000 rpm and twice for 2 min at 4,000 rpm, cooled on ice between the cycles. The samples were subsequently centrifuged for 2 min at 10,000 g and purified in TRIzol/chloroform (5:1) and chloroform. For RNA precipitation, the samples were incubated in isopropanol at −20°C overnight and centrifuged for 30 min at 10,000 g. RNA samples were washed in ethanol and resuspended in 30 μl of RNase-free water. Residual DNA in the RNA samples was removed by DNase I treatment (Ambion). Typically, a concentration between 1 and 3.5 μg/μl was obtained. RNA quality was checked on a 1% agarose gel.

### 5′ and 3′ race

**Figure 1:**
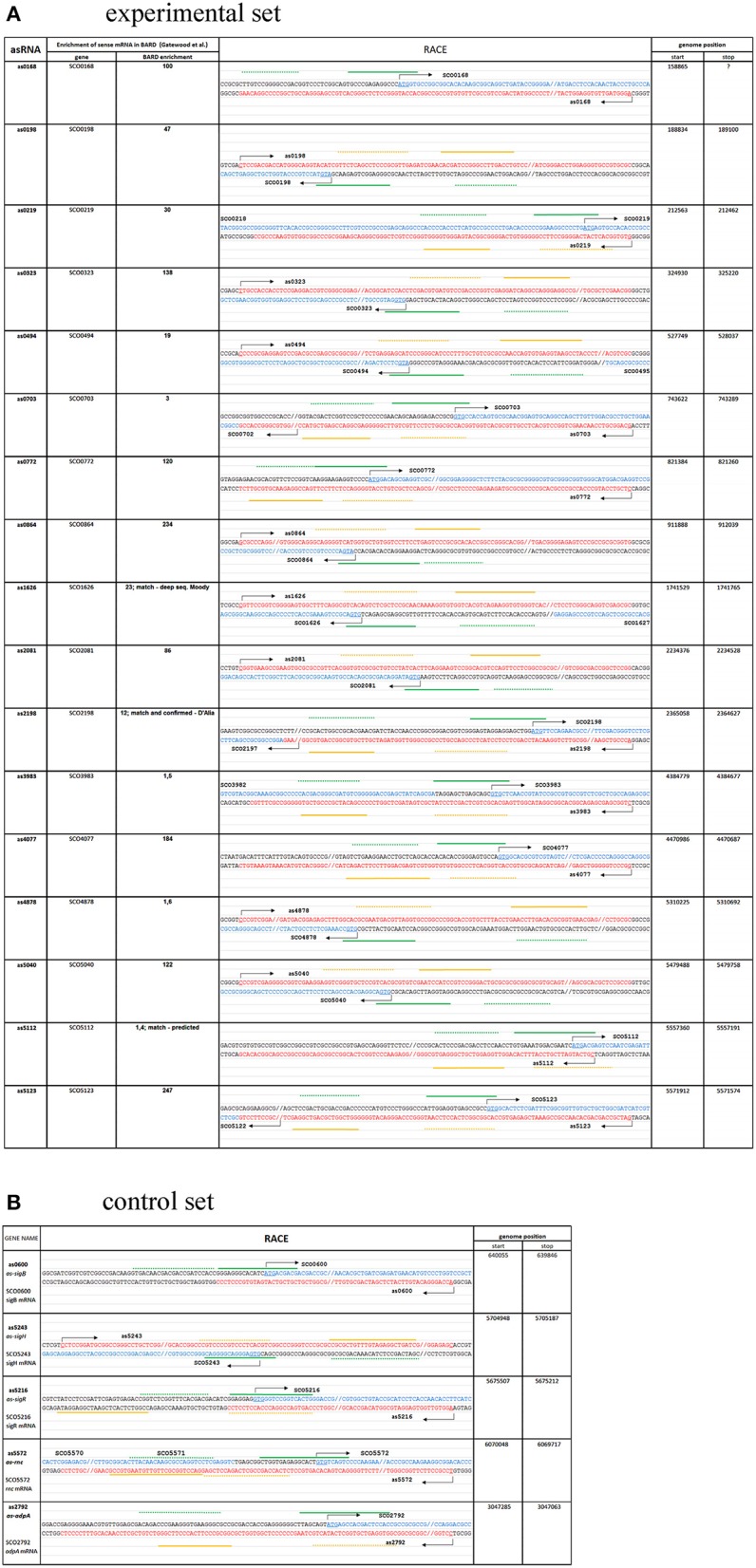
Novel asRNAs revealed by 5′and 3′RACE method, and their genome locations (sequence of asRNA in red, sequence of mRNA in blue). Transcriptional start sites are indicated by arrows. Full green line represents 5′RACE inner primers, dotted green line represents 5′RACE outer primers, full orange line represents 3′RACE inner primers, dotted orange line represents 3′RACE outer primers. **(A)** Experimental set; **(B)** Control set; (see text for details).

RNA samples were isolated after 48 h of cultivation of both wt and *rnc* strains. Antisense RNA expressions were tested by means of the FirstChoice RLM-RACE Kit (Ambion) following the manufacturer's protocol with the several exceptions:

1. Because the uncapped 5′ ends of bacterial RNAs are sensitive to the CIP (calf intestinal phosphatase) enzyme dephosphorylation, the treatment was omitted from the 5′ RACE procedure.
2. A gene-specific primer (see Figure 1) was used instead of random decamers in the 5′RACE. The 5′RACE primers as well as the probes used for Northern blot hybridizations were designed to cover the ribosome binding site and start codon of a cognate mRNA. All of the primers were designed using the Primer3 software (http://sourceforge.net/projects/primer3/) (Untergasser et al., 2012).
3. The PrimeScript (Takara, 100 units per 10 μl of reaction mixture) reverse transcriptase was always included in the experiment. Negative control lacking the enzyme was always attached to the experiment.
4. The reverse transcription proceeded at 42°C for 45 min and 48°C for 10 min.
5. Preceding the 3′RACE, total RNA samples were polyadenylated by 5 units of Poly(A) Polymerase I (New England Biolabs), according the manufacturer's protocol.

Final PCR products were separated on a 1.2% agarose gel. Products that were found in samples but absent in negative controls were excised and purified using the Qiagen MinElute PCR purification kit. The purified products were cloned into the TOPO vector using the TOPO TA Cloning (Invitrogen) and transformed into *E. coli* One Shot TOP10F' competent cells (Invitrogen). Plasmids containing the cDNA inserts were extracted using the QIAprep Miniprep kit, and sequenced to map 5′ and 3′ ends of RNAs reverse-transcribed.

### Reverse transcription and PCR

Experiments were performed according to the reverse transcription and PCR protocols described in the FirstChoice RLM-RACE Kit using gene specific DNA probes (details in Results and discussion). The PrimeScript (Takara, 100 units per 10 μl of reaction mixture) was used as a reverse transcriptase. A negative control PCR reaction used the original RNA sample as a template.

#### Northern blot analysis

RNA samples (30 μg) were denatured for 10 min at 70°C in RNA loading buffer (95% formamide, 0.1% bromophenol blue, 0.1% xylene cyanol, 10 mM EDTA) and separated in a 1% agarose gel containing formaldehyde, provided by the NorthernMax Kit (Ambion). Separated samples were transferred onto positively charged nylon membranes (ZetaProbe, Bio-Rad) by electroblotting at 240 mA for 45 min. The nylon membrane was UV-crosslinked.

Oligonucleotides were radioactively labeled on their 5′ ends by γ-^32^P-ATP using T4 polynucleotide kinase (Thermo Scientific) and purified (QIAquick Nucleotide Removal Kit, Qiagen). Hybridization was performed in ULTRAhyb hybridization buffer (Ambion) overnight at 37–42°C. The membranes were then washed twice with 2xSSC, 0.1% SDS (NorthernMax kit) at room temperature and once with 0.1xSSC, 0.1% SDS (NorthernMax kit) at 42°C. The membranes were dried and exposed in a BAS cassette on the imaging plate (Fuji-Film) for 4 days. The signals were visualized using a Phosphorimager FX (Bio-Rad) and quantified using QuantityOne analysis software (Bio-Rad), where the signals were standardized proportionally to the 5S RNA levels. Each northern blot was performed at least twice with samples from separate cultivations in the same conditions.

## Results

Using the RNA-seq approach, Gatewood (Gatewood et al., 2012) compared gene expression between the *S. coelicolor* M145 wild type strain and the JSE1880 *rnc*-mutant strain. The authors found that approximately 10% of all mRNAs from the vegetative state of growth were directly or indirectly affected by RNase III. In addition, they applied RNA immunoprecipitation to detect mRNAs targeted by the enzyme (referred to in their paper as BARD, bead-antibody-RNA-D70A strain). However, the necessity of involvement of other transcripts for the binding of the double-strand-specific enzyme to the mRNAs is still unknown in the case of *Streptomyces*. *In vitro* assays showed that the RNase III digests mRNA transcripts SCO3982 to SCO3988 and SCO5737 unattended (i.e., without asRNA) (Gatewood et al., 2012), whereas another unattended transcript, SCO0762, was not cleaved (Xu et al., 2008). Although *Streptomyces* as GC rich bacteria form highly structured RNAs, naturally occurring stem-loop structures of most mRNAs are too short to be bound by the RNase III enzyme that requires a minimum of approximately 20 bp of double stranded RNA for binding *in vivo* (Robertson, 1982).

### RNase III-binding mRNAs and small RNAs genome vicinities

Here, to probe if the antisense RNA expression occurs in the vicinity of the RNase III-binding mRNAs (or alternatively, the binding is not complexed with asRNA), we firstly analyzed the genes whose transcripts are affected by RNase III (i.e., those mRNAs that are increased by more than two-fold in the JSE1880 *rnc-*mutant or detected in BARD as listed in the Table S2 in Gatewood; Gatewood et al., 2012). We asked whether in the proximity of these genes lies a gene encoding for any of 1713 small RNAs found or predicted in *S. coelicolor* up-to-date (Pánek et al., 2008; Swiercz et al., 2008; D'Alia et al., 2010; Vockenhuber et al., 2011; Moody et al., 2013) that could act on the messenger by the antisense mechanism. We found out that from the 153 mRNAs increased in JSE1880 (at a single experimental time point), 45 neighbor with one of the 1713 small RNAs that could thus theoretically act as an asRNA. From these 45 mRNAs, 21 mRNAs were listed in the BARD, i.e., they bind RNase III. These data, summarized in Table 1, encouraged us to search for novel asRNAs expressed in the opposite direction to other RNase III-binding mRNAs.

**Table 1:** *In silico* search for sRNA genes adjacent to mRNAs that are up-regulated in rnc mutant.

**Selected genes whose expression increased in JSE1880 (blue indicate transcripts present in the BARD; Gatewood et al.**, **2012****)**		**Antisence/adjacent small RNAs (scr = small coelicolor RNA)**		**References**	Relative orientation of asRNA gene to its possible mRNA target (red arrow) and the second adjacent gene (black arrow), ^*^ unknown (ambiguous) strand of asRNA gene, = > orientation of asRNA gene, cutoRNA—antisense transcripts acting on messenger's 3′ end		**Comment**
SCO	0499	scr	0500	Moody et al., 2013	Ambiguous	–> ^*^ <–	mRNA up-regulated in JSE1880 (Gatewood et al., 2012)
SCO	0500	scr	0500	Moody et al., 2013	Ambiguous	–> ^*^ <–	mRNA up-regulated in JSE1880 (Gatewood et al., 2012)
SCO	1150	scr	1150	Moody et al., 2013	cutoRNA	–> <= <–	mRNA up-regulated in JSE1880 (Gatewood et al., 2012)
SCO	1565	scr	1566	Swiercz et al., 2008	Ambiguous	<– ^*^ –>	Predicted
SCO	1626	scr	1625	Moody et al., 2013	cutoRNA	–> <= <–	as1625 analyzed here
SCO	1630	scr	1631	Swiercz et al., 2008	Ambiguous	<– ^*^ <–	Predicted
SCO	1659	scr	1659	Swiercz et al., 2008	Ambiguous	–> ^*^ –>	Predicted
SCO	1700	scr	1700	D'Alia et al., 2010	cis asRNA	<– = > <–	Predicted by RNAz (D'Alia et al., 2010)
SCO	1906	scr	1907	Swiercz et al., 2008	Ambiguous	<– ^*^ <–	Predicted
SCO	2197	scr	2198	Swiercz et al., 2008	Ambiguous	<– ^*^ –>	Predicted
SCO	2198	scr	2198	D'Alia et al., 2010	cis asRNA	<– <= –>	Confirmed, *as2198* analyzed here
SCO	3003	scr	3004	Swiercz et al., 2008	Ambiguous	<– ^*^ –>	Predicted
SCO	3113	scr	3114	Swiercz et al., 2008	Ambiguous	–> ^*^ <–	Predicted
SCO	3132	scr	3133	D'Alia et al., 2010	cis asRNA	–> <= <–	Predicted by RNAz (D'Alia et al., 2010)
SCO	3216	scr	3216	Swiercz et al., 2008	Ambiguous	<– ^*^ –>	Predicted
SCO	3217	scr	3217	Swiercz et al., 2008	Ambiguous	–> ^*^ –>	Predicted
SCO	4095	scr	4096	Swiercz et al., 2008	Ambiguous	–> ^*^ –>	Predicted
SCO	4142	scr	4143	Swiercz et al., 2008	Ambiguous	<– ^*^ <–	Predicted
SCO	4144	scr	4145	Swiercz et al., 2008	Ambiguous	<– ^*^ <–	Predicted
SCO	4145	scr	4145	Swiercz et al., 2008	Ambiguous	<– ^*^ <–	Predicted
SCO	4145	scr	4146	Swiercz et al., 2008	Ambiguous	<– ^*^ –>	Predicted
SCO	4229	scr	4229	Swiercz et al., 2008	ambiguous	<– ^*^ –>	Predicted
SCO	4249	scr	4249	Swiercz et al., 2008	Ambiguous	<– ^*^ –>	Predicted
SCO	4283	scr	4283	Moody et al., 2013	cutoRNA	–> <= <–	mRNA up-regulated in JSE1880 (Gatewood et al., 2012)
SCO	4698	scr	4699	Moody et al., 2013	Ambiguous	–> <= –>	Predicted
SCO	4748	scr	4749	Moody et al., 2013	cutoRNA	–> = > <–	mRNA up-regulated in JSE1880 (Gatewood et al., 2012)
SCO	4882	scr	4883	Swiercz et al., 2008	Ambiguous	–> ^*^ –>	Predicted
SCO	4947	scr	4947	Swiercz et al., 2008	Ambiguous	–> ^*^ –>	Predicted
SCO	5106	scr	5106	Moody et al., 2013	cutoRNA	–> = > <–	mRNA up-regulated in JSE1880 (Gatewood et al., 2012)
SCO	5112	scr	5112	Swiercz et al., 2008	cis asRNA	–> ^*^ –>	Predicted, *as5112* analyzed here
SCO	5142	scr	5143	Moody et al., 2013	Ambiguous	–> <= –>	Predicted
SCO	5145	scr	5145	Swiercz et al., 2008	Ambiguous	<– ^*^ –>	Predicted
SCO	5145	scr	5146	Moody et al., 2013	cutoRNA	–> = > <–	mRNA up-regulated in JSE1880 (Gatewood et al., 2012)
SCO	5163	scr	5164	Swiercz et al., 2008	Ambiguous	<– ^*^ –>	Predicted
SCO	5476	scr	5476	Swiercz et al., 2008	Ambiguous	–> ^*^ –>	Predicted
SCO	5519	scr	5518	Moody et al., 2013	Ambiguous	<– ^*^ –>	Predicted
SCO	5520	scr	5521	Swiercz et al., 2008	Ambiguous	–> ^*^ –>	Predicted
SCO	5521	scr	5521	Swiercz et al., 2008	Ambiguous	–> ^*^ –>	Predicted
SCO	5536	scr	5536	Pánek et al., 2008	Ambiguous	<– ^*^ –>	Termed #234
SCO	5537	scr	5537	Swiercz et al., 2008	Ambiguous	–> ^*^ <–	Predicted
SCO	5757	scr	5756	Swiercz et al., 2008	Ambiguous	–> ^*^ –>	Predicted
SCO	6277	scr	6277	Moody et al., 2013	cis asRNA	–> <= –>	mRNA up-regulated in JSE1880 (Gatewood et al., 2012)
SCO	6283	scr	6284	Moody et al., 2013	Ambiguous	–> ^*^ –>	Predicted
SCO	6284	scr	6284	Moody et al., 2013	Ambiguous	–> ^*^ –>	Predicted
SCO	6284	scr	6285	Moody et al., 2013	Ambiguous	–> <= –>	Predicted
SCO	6396	scr	6396	Moody et al., 2013	Ambiguous	–> ^*^ –>	Predicted
SCO	6716	scr	6716	Moody et al., 2013	cutoRNA	–> <= <–	mRNA up-regulated in JSE1880 (Gatewood et al., 2012)
SCO	6716	scr	6717	Moody et al., 2013	cutoRNA	–> = > <–	–
SCO	6728	scr	6729	Moody et al., 2013	cutoRNA	–> = > <–	mRNA up-regulated in JSE1880 (Gatewood et al., 2012)

### Experimental search for novel *cis*-antisense transcripts

Genes for the experimental analyses had been selected independently of the *in silico* search above. We altogether tested 30 genes for the possible novel antisense expression. The initial experimental set consisted of 17 exemplar genes which we selected out of a total of 37 genes whose mRNAs were enriched in the BARD, i.e., they co-precipitated with RNase III (listed in Table 2, see also Gatewood's Table 2 and Table S3 in Gatewood et al., 2012). Within this set, only *as1625, as2198*, and *as5112* genes have been predicted before (see Table 1). The control set consisted of three other genes—SCO5737, *adpA*, and *rnc* (the gene of RNase III) that had been previously shown to be directly targeted by RNase III *in vitro* (Xu et al., 2008, 2010; Gatewood et al., 2012). Additionally, to determine whether the existence of antisense transcripts involves only those messengers bound by RNase III or is even more widespread, we decided to add into the control set the genes whose transcripts have not been shown to bind RNase III. For these tests, we selected 10 genes encoding sigma factors (HrdA, HrdB, HrdC, HrdD, SigB, SigD, SigE, SigH, SigR, and WhiG) as important transcriptional regulators that govern gene expression, controlling cell development and/or responses to various stresses (Bobek et al., 2014).

**Table 2:** The list of analyzed genes.

	**Genes selected for the analyses**		**Encoded protein**	**Relation of mRNA to RNase III**		**RACE**			
				**Ratio enrichment in BARD**	**Fold increase in JSE1880**	**5′/3′ end detection result**	**Original strain from which the final RACE data were determined**	**Northern blot detection result**	5′end-antisense expression independently confirmed by RNA-seq data^*^
				**Data taken from Gatewood et al. (****2012****)**					
**Experimental set (mRNA co-precipitated with RNase III** ***in vivo*****)**	SCO	0168	Possible regulatory protein	100	2.41	+/−	WT	–	+/+
	SCO	0198	Hypothetical protein	47.6	2.70	+/+	WT	+	−/−
	SCO	0219	Putative nitrate reductase delta chain	30	2.18	+/+	WT, RNC	+	−/−
	SCO	0323	Hypothetical protein	138	1.62	+/+	WT, RNC	+	+/+
	SCO	0494	Probable iron-siderophore binding lipoprotein	19	−2.14	+/+	RNC	+	−/−
	SCO	0703	Putative regulatory protein	119	1.36	+/+	RNC	+	−/−
	SCO	0772	Putative regulatory protein	3	2.46	+/+	WT, RNC	+	+/−
	SCO	0864	Probable ECF-family sigma factor	234	1.70	+/+	WT	+	−/−
	SCO	1626	Cytochrome P450	22.8	−2.6	+/+	WT, RNC	+	+/+
	SCO	2081	Hypothetical protein	86	1.23	+/+	WT, RNC	–	−/−
	SCO	2198	Glutamine synthetase I	12.4	2.9	+/+	RNC	+	+/+
	SCO	3983	Hypothetical protein	1.5	2.14	+/+	WT, RNC	+	+/−
	SCO	4077	Hypothetical protein	184	−1.01	+/+	WT, RNC	+	+/−
	SCO	4878	Glycosyltransferase	1.6	2.00	+/+	RNC	–	+/−
	SCO	5040	Hypothetical protein	122	1.22	+/+	RNC	–	−/−
	SCO	5112	Putative ABC transport systém integral membrane protein, BldKA	1.4	2.56	+/+	WT, RNC	+	+/−
	SCO	5123	Small membrane protein	247	1.24	+/+	WT, RNC	+	+/−
**Control set**	SCO	2792	AdpA	*In vitro* binding		+/+	RNC	+	+/+
	SCO	5572	RNase III	*In vitro* binding		+/+	WT	+	+/+
	SCO	5737	Guanosine pentaphosphate synthetase/polyribonucleotide nucleotidyltransferase	*In vitro* binding		−/−	–	–	+/+
	SCO	0600	RNA polymerase sigma factor SigB	Unknown		+/+	WT	+	+/−
	SCO	0895	RNA polymerase sigma factor HrdC	Unknown		−/−	–	–	+/−
	SCO	2465	RNA polymerase sigma factor HrdA	Unknown		−/−	–	–	+/+
	SCO	3202	RNA polymerase sigma factor HrdD	Unknown		−/−	–	–	+/+
	SCO	3356	RNA polymerase sigma factor SigE	Unknown		−/−	–	–	+/−
	SCO	4769	RNA polymerase sigma factor SigD	Unknown		−/−	–	–	+/−
	SCO	5216	RNA polymerase sigma factor SigR	Unknown		+/+	WT	+	+/−
	SCO	5243	RNA polymerase sigma factor SigH	Unknown		+/+	WT	+	+/+
	SCO	5621	RNA polymerase sigma factor WhiG	Unknown		−/−	–	–	−/−
	SCO	5820	RNA polymerase sigma factor HrdB	Unknown		−/−	–	–	+/−

* *The expression was confirmed from the RNA-Seq data (Vockenhuber et al., 2011)/(Moody et al., 2013)*.

We reasoned that many asRNAs overlap the ribosome binding site and the start codon of their target, possibly leading to negative translational control. Following this, all of the DNA primers used here to find antisense transcripts have been designed accordingly (Figure 1). To demonstrate expression of antisense transcripts, 5′ RACE analyses were performed using the primers. Sequence(s) extended from the primer was/were PCR amplified and sequenced. To avoid false positive results, each reverse transcription was accompanied with a negative control sample lacking the reverse transcriptase in the reaction. The resulting electrophoretograms of both the experimental and the negative control samples were compared to exclude non-unspecific fragments in the experimental sample from sequencing.

From the total of 30 samples tested (involving both the experimental and the control set), 22 revealed a novel antisense transcript in *cis* (5′ end(s) detected; see Table 2).

From the 17 samples of the experimental set (i.e., our selection from those mRNAs enriched in the BARD), 17 cognate asRNAs were detected (three of them have been predicted previously as could be seen in Table 1), signifying a 100% outcome within this group (Figure 1A). The 3′ RACE revealed 16 3′ ends within the experimental set (*scr0168* unsuccessful, northern blot was not performed).

In the control set (Figure 1B), our RACE data detected transcripts antisense to *adpA* (*as-adpA*), *rnc* (*as-rnc*), and three sigma factor genes (*as-sigB, as-sigH, as-sigR*). Seven sigma factor genes and the transcript SCO5737, which is bound by RNase III *in vitro*, did not reveal antisense expression.

Due to our primer design, we did not detect such antisense transcripts acting on messenger's 3′ end [in Moody (Moody et al., 2013) termed as cutoRNAs]. Also the experimental approach did not allow identification of *trans*-encoded sRNAs, although similar complementary sequence on the genome could be found (as examples, novel RNAs as0772, as0600, as3983, and as5216 could theoretically act on other targets throughout the genome).

**Figure 2:**
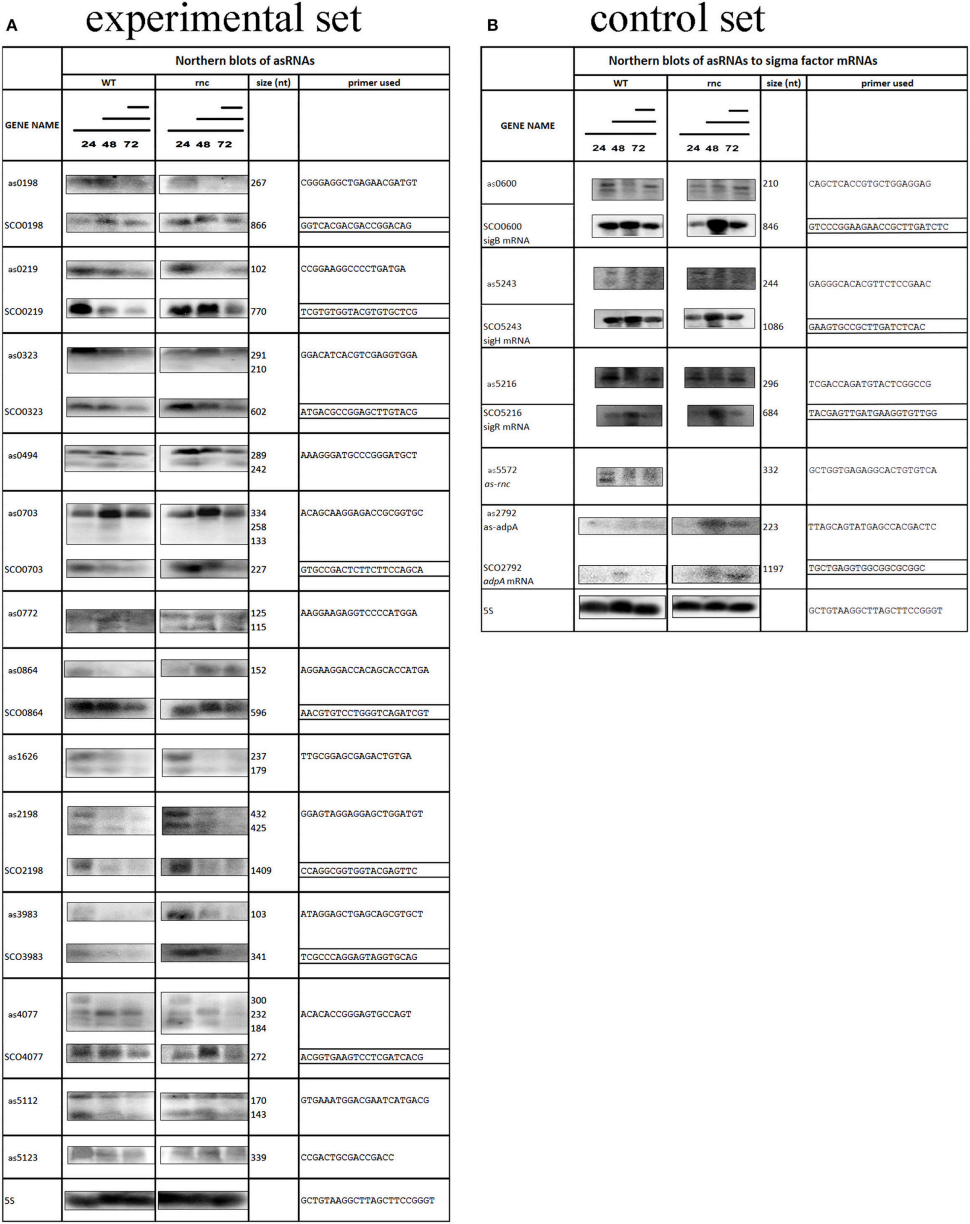
Differential expression analyses of novel asRNAs and their target mRNAs in WT and rnc strains. Three black lines (from long to short) represent RNA samples from vegetative mycelium (24 h), aerial mycelium (48 h), and spores (72 h), respectively. To enable comparison of the expression profiles, all asRNA-mRNA pairs from both WT and rnc strains were analyzed on the same blot. Sizes of the products well corresponded to those obtained by RACE. Primers used are shown on the right. The 5S loading control is included below. The signal quantification is presented in Table S2. **(A)** Experimental set; **(B)** Control set; (see text for details).

Sometimes the RACE analysis may produce false positive results due to the RNA self-priming during the reverse transcription which could be theoretically caused by stem-loop structures formation. In order to confirm the existence of the newly found *cis-*antisense transcripts and to compare their expression during *Streptomyces* cell development (24, 48, and 72 h at standard growth conditions, see methods), we performed northern blot analyses. From the total of 22 analyzed, 18 asRNAs revealed an apparent signal (Figure 2; for the raw northern blot images see Presentation 1 in Supplementary Material). The signals of the remaining four were too weak or undetectable.

Details describing expression profiles of asRNAs are summarized in Table S1 and signal quantification is presented in Table S2 online, where the listed relative values proportional to the 5S RNA controls correspond with intracellular levels of the analyzed transcripts.

### *Vice versa: scr2101* small RNAs found in BARD (Gatewood et al., 2012) is antisense to the SCO2100-SCO2101 transcript

In Gatewood's work, expression of two small RNAs, *scr6925* and *scr2101*, was enriched in the *rnc* strain (scr = small *S. coelicolor* RNA). These data imply their antisense role and subsequent processing by the RNase III enzyme. The expression of *scr2101* was previously revealed by Swiercz (Swiercz et al., 2008). Gatewood et al. (2012) showed that the molecule is up-regulated in the JSE1880 strain (lacking the RNase III), where its level was 7-fold higher compared to that in the wild type strain. *scr2101* was also shown to be bound by the enzyme (presented in the BARD, although its enrichment was only 0.57). As the RNA's gene lies on the opposite strand between SCO2100 and SCO2101, we raised the question whether these two protein-coding genes form an operon, producing one common transcript targeted by the *scr2101* antisense RNA. The potential SCO2100-SCO2101 common transcript was used here as a template for reverse transcription using a DNA probe complementary to the 5′ end of the SCO2101 mRNA (TGTCCCGGCTGCTCCAGGGA). The second DNA primer, used for the following PCR amplification, was identical to the 3′ end of the SCO2100 mRNA sequence (CGTAGGTCCCCGCCCGCT), thus forming a 635-nt product, which was indeed produced. Use of a negative control PCR reaction, where the original RNA was used instead of a template, eliminated the risk of false positive results (Figure S1). This finding suggests that the antisense function of *scr2101* (which thus should be termed as *as2101*) targets the region between two open reading frames. Although the binding experiments are outside the scope of this paper, our findings raise demand to expand the RNase III-binding analyses to the unannotated sRNA transcriptome.

## Discussion

### Novel asRNAs to mRNAs that bind RNase III *in vitro*

The double stranded stems of stem-loop mRNA structures are thought to be too short to be digested by the RNase III enzyme (Robertson, 1982). On the other hand, one may argue that the stem-loop structures on several mRNAs are the only possible targets for RNase III activity. Indeed, the enzyme is involved in pre-rRNA, tRNA, and polycistronic RNA processing (Conrad and Rauhut, 2002; Drider and Condon, 2004), where such stem-loop structures are present and possibly long enough to be cleaved by the enzyme. Gatewood clearly showed that some lone mRNAs are targeted and cleaved by RNase III *in vitro* (SCO5737, or the SCO3982 to SCO3988 mRNA region). In accordance to the first example, our experiments did not reveal any antisense transcript to the SCO5737 (which encodes a polynucleotide phosphorylase). Its mRNA transcript is 2,220 nt in length, and thus might be capable of forming several longer double-stranded regions observed by RNAfold (http://rna.tbi.univie.ac.at/cgi-bin/RNAWebSuite/RNAfold.cgi; fig. not shown), which are most likely targeted by the RNase III enzyme *in vitro*.

On the other hand, we show that at least one member of the SCO3982-SCO3988 operon – SCO3983 possesses an antisense gene. Its transcript, *as3983*, is 103 nt long and exhibited a strongest expression signal in vegetative cells (24 h) of the *rnc* strain. The mRNA also had a strong signal at 24 h and 48 h, even elevated in the *rnc* strain. Moreover, we observed that the *as3983* sequence is nearly identical to the region adjacent to SCO3268 and theoretically may thus also act on this transcript in *trans*.

In addition to the identified as3983 RNA, we have found antisense transcripts to two other mRNAs targeted by RNase III *in vitro*. These two mRNAs code important developmental regulators that influence antibiotic production—AdpA and the RNase III itself.

According to Gatewood's results, the *adpA* mRNA was enriched 22-fold in the BARD. Therefore, we asked whether AdpA expression is controlled by an antisense mechanism. Indeed, our RACE analysis revealed a 223-nt long antisense transcript termed *as-adpA* (Figure 1B) and although subsequent northern blot weakly detected the transcript in the wt strain (faint signal at 72 h stage of growth, Figure 2B), it was clearly found in the *rnc* strain at later stages, peaking at the 48 h old mycelium.

The *rnc* gene (SCO5572) that encodes the RNase III enzyme is the last member of a three gene operon, about 2,000 nt in length, that also encodes a hypothetical protein SCO5570 and a ribosomal protein L32 (SCO5571). A point mutation at amino acid position 120 of the RNase III protein causes deficiency in the ribonucleolytic activity of the enzyme, whereas its ability to bind double stranded RNAs remains intact (Huang et al., 2005). Observations that the *rnc* mRNA abundance is increased in these mutants led to discovery that RNase III cleaves among others its own transcript (Xu et al., 2008). Consistently, our northern blot analyses did not detect the *rnc* transcript in the wt strain, though we clearly confirmed the *as-rnc* RNA, which was detectable during the time course. Hence the antisense mechanism is probably involved in gene expression control of regulatory proteins such as the RNase III enzyme or the AdpA transcription regulator. Our future effort will be focused on more detailed characterization of the involvement of these two novel asRNAs in *Streptomyces* development.

### *as2198* RNA as an example from the experimental set

*as2198* is an asRNA to the *glnA* gene (SCO2198, encoding a glutamine synthase) and was previously independently shown to be expressed, termed *cnc2198.1* (D'Alia et al., 2010). The authors performed a detailed functional analysis and revealed that overexpression of *cnc2198.1* affects growth rate and antibiotic production. In the overexpression strain, the intracellular level of the targeted GlnA protein was decreased by 40%. The authors speculated that the *glnA-cnc2198.1* RNA complex blocks *glnA* translation, which may lead to its subsequent degradation. We further hypothesize that the complex is degraded by the RNase III enzyme, as our northern blot revealed that the asRNA level is increased in the *rnc* mutant. Our RACE mapping estimated the size of the antisense transcript to be 432 nt, nearly four times longer than the 121 nt transcript described by D'Alia et al. (2010). Moreover, the detected 3′ end overlapped the adjacent SCO2197, revealing that *as2198* is in fact the 5′UTR of the SCO2197 mRNA. The fact that our northern blot showed a second, weaker fragment of 425 nt in size, could mean that the SCO2197 gene possesses another promoter or its transcript is further processed. Both of the detected fragments, as well as the cognate SCO2198 mRNA, had the strongest expression signals in samples from vegetative 24-h old cells, even strengthened in the *rnc* strain.

### Are the sense-antisense transcripts cleaved by RNase III?

Because the sense mRNAs of the antisense transcripts found here are targeted by RNase III (or at least, their expression was negatively affected in the presence of the enzyme), we speculated that the RNase III enzyme is the most likely candidate for the paired transcript degradations. The 18 northern blot positive results proved the existence of novel asRNAs. However, only five of those asRNAs were increased in the *rnc* strain when compared to the expression in the wild type strain. These included *as0494, as0864, as2198, as3983*, and the *as-adpA*. On the other hand, in two other cases, our northern blots suggest even a positive effect of the presence of RNase III enzyme on the cellular level of asRNAs (*as0323* and *as5112*, see below). Consistently, Gatewood (Gatewood et al., 2012) showed that the majority of known sRNAs, detected in their RNA-seq analysis, do not exhibit significant expression differences between the wild-type and the *rnc*-deletion mutant. As another example of sense-antisense RNA pair with an undistinguishable or even a positive effect of the enzyme on its stability in *Streptomyces* is the *scr4677*-SCO4676 complex (Hindra et al., 2014). The possibility that the RNase III enzyme does not always post-transcriptionally degrade sense-antisense complexes is also inferred from a study on sRNA degradation by three different RNases (RNase Y, J1, and III) in another Gram-positive bacterial model, *Bacillus subtilis* (Durand et al., 2012). In *Bacillus*, RNase III depletion has little effect on antisense RNAs observed by high-resolution tiling arrays. Although several RNAs showed increased abundance in the RNase III mutant, their half-lives were not affected by the enzyme, as observed by northern blot analysis. The authors concluded that the role of RNase III in *Bacillus subtilis* lies more likely in indirect transcriptional control rather than post-transcriptional RNA turnover. One may argue that the function of the enzyme might be substituted by other ribonucleases in *rnc* mutants. As the RNase III-binding mRNAs may serve as a fruitful source for novel antisense transcript discoveries, we can assume that the mRNA-asRNA pairs are targeted by the enzyme but not always degraded. An involvement of some other RNA-binding proteins, such as RNA helicase or Hfq-like protein which has not been found in *Streptomyces* yet, could be expected (Gerhart Wagner, Uppsala University, Sweden, personal communication). On the other hand, the possibility that in other cases the antisense transcripts might protect their mRNA targets against the RNase III cleavage should be taken in mind. Clarification of the exact role of RNase III enzyme in *Streptomyces* remains to be established.

### asRNAs as a potential part of the gene expression control system in *Streptomyces*

The genus *Streptomyces* can be presented as a model bacterial group lying on the top of prokaryotic cellular complexity. Their 8–10 Mbp long genome encodes all the developmental stages, including morphological changes (spore formation and germination, vegetative branching hyphae, aerial twisting mycelium), secondary metabolite production (antibiotics and a variety of other bioactive compounds, siderophores, pigments, etc.) and a capacity to respond to all possible environmental changes (diverse stresses) that are encountered. Developmental transitions and environmental intricacy require advanced regulatory networks that involve a concerted action of more than 900 transcription regulatory proteins known thus far. Here we unveiled 22 novel antisense transcripts, out of which 18 were confirmed by northern blot analyses. These results suggest an equivalent role and possibly even bigger number of non-protein-coding RNA regulators. The *cis*-antisense transcripts are efficient gene expression modulators with minimal space requirements on their genome (Georg and Hess, 2011), with a theoretical capability to act on nearly all genes. Moreover, the mode of their action (whether based on co-transcriptional collision or post-transcriptional 100% complementarity) is simple and effective. Our work suggests that antisense transcription is widespread in *Streptomyces* and somehow connected with the function of RNase III, although the absence of the *rnc* gene did not greatly influence the majority of the transcripts. It is noteworthy that the majority of antisense transcripts found here escaped previous sRNA predictions and/or whole genome searches, suggesting that their expression level is often low and may be lost during statistical background subtraction. Nevertheless, the results in this work confirm the extensiveness of the antisense transcripts and raise the demand for elucidation of their role in gene expression control in *Streptomyces*.

## Author contributions

DŠ, PP, IM, and KŠ conceived the RACE and northern blot experiments, JB performed the *in silico* analysis and provided the project design, analyzed the results and wrote the manuscript. All authors reviewed the manuscript.

### Conflict of interest statement

The authors declare that the research was conducted in the absence of any commercial or financial relationships that could be construed as a potential conflict of interest.

## Supplementary material

Figure S1 SCO2100-SCO2101 RT-PCR. RNA samples were isolated from both wt (lane 1) and rnc (lane 2) 48 h old cultures from the R2YE medium. The first probe TGTCCCGGCTGCTCCAGGGA primed the reverse transcription reaction and together with the second probe CGTAGGTCCCCGCCCGCT was used in the subsequent PCR. Additional samples originated from both strains but lacking reverse transcriptase in the reaction were used as negative controls (lanes 3 and 4).

Table S1 Description of the expression profiles of detected asRNAs.

Table S2 Quantification of the northern blot signals presented in Figure 2.

Table S2 Raw northern blot images.
