# Supplementary figures and images for: RNase III-Binding-mRNAs Revealed Novel Complementary Transcripts in Streptomyces
Source: Front Microbiol. 2018 Jan 15;8:2693. doi: 10.3389/fmicb.2017.02693 (PMC5775266; doi:10.3389/fmicb.2017.02693)

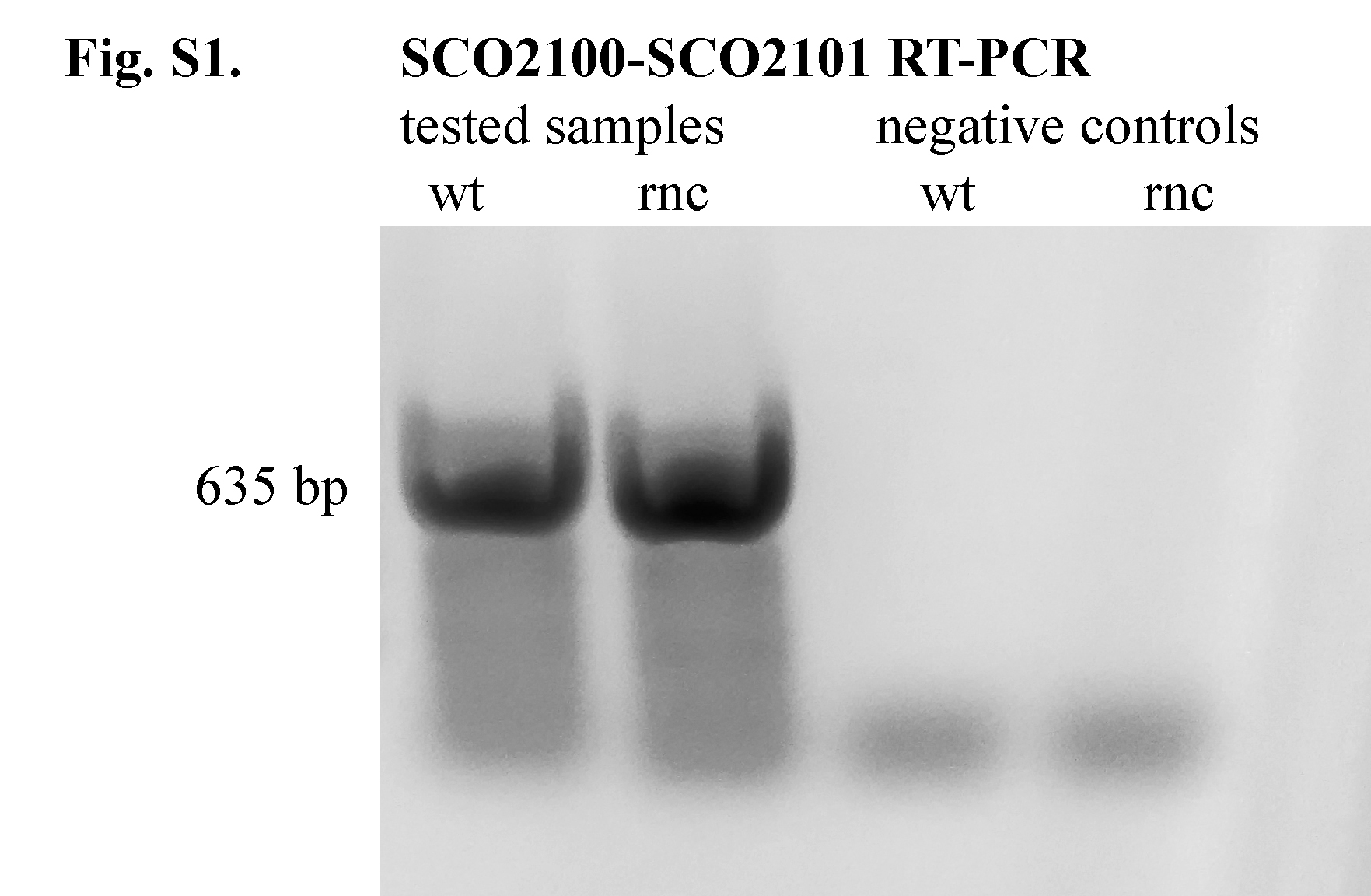

Supplement: Figure S1 — SCO2100-SCO2101 RT-PCR. RNA samples were isolated from both wt (lane 1) and rnc (lane 2) 48 h old cultures from the R2YE medium. The first probe TGTCCCGGCTGCTCCAGGGA primed the reverse transcription reaction and together with the second probe CGTAGGTCCCCGCCCGCT was used in the subsequent PCR. Additional samples originated from both strains but lacking reverse transcriptase in the reaction were used as negative controls (lanes 3 and 4). [file Image1.JPEG]
