# Supplementary material for: RNase III-Binding-mRNAs Revealed Novel Complementary Transcripts in Streptomyces
Source: Front Microbiol. 2018 Jan 15;8:2693. doi: 10.3389/fmicb.2017.02693 (PMC5775266; doi:10.3389/fmicb.2017.02693)

RNase III-binding-mRNAs revealed novel complementary transcripts in *Streptomyces*

Dita Šetinová , Klára Šmídová[1], Pavel Pohl, Inesa Music, and Jan Bobek

raw northern blot images

Supplementary File

as0198

rnc 24h, 48h, 72h; wt 24h, 48h, 72h

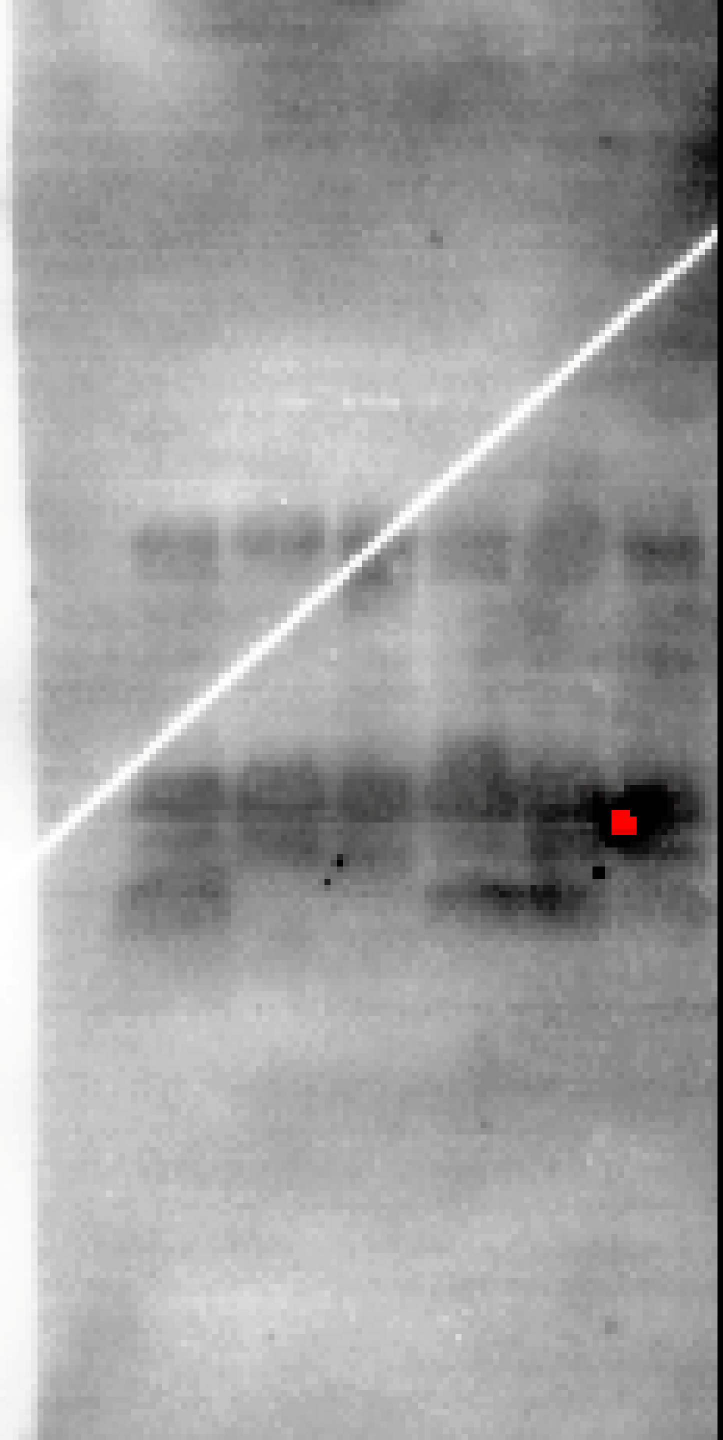

as0219

rnc 24h, 48h, 72h; wt 24h, 48h, 72h

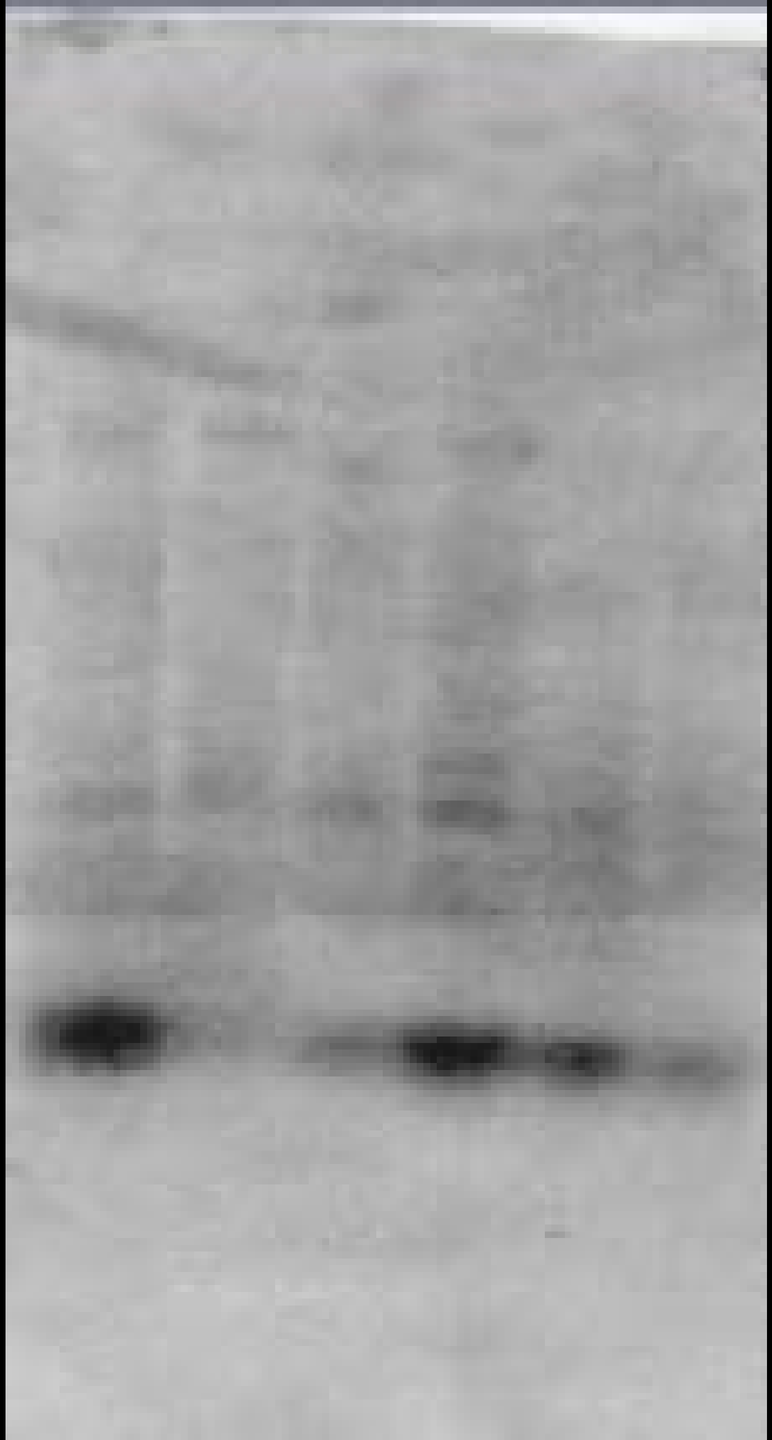

as0323

rnc 24h, 48h, 72h; wt 24h, 48h, 72h

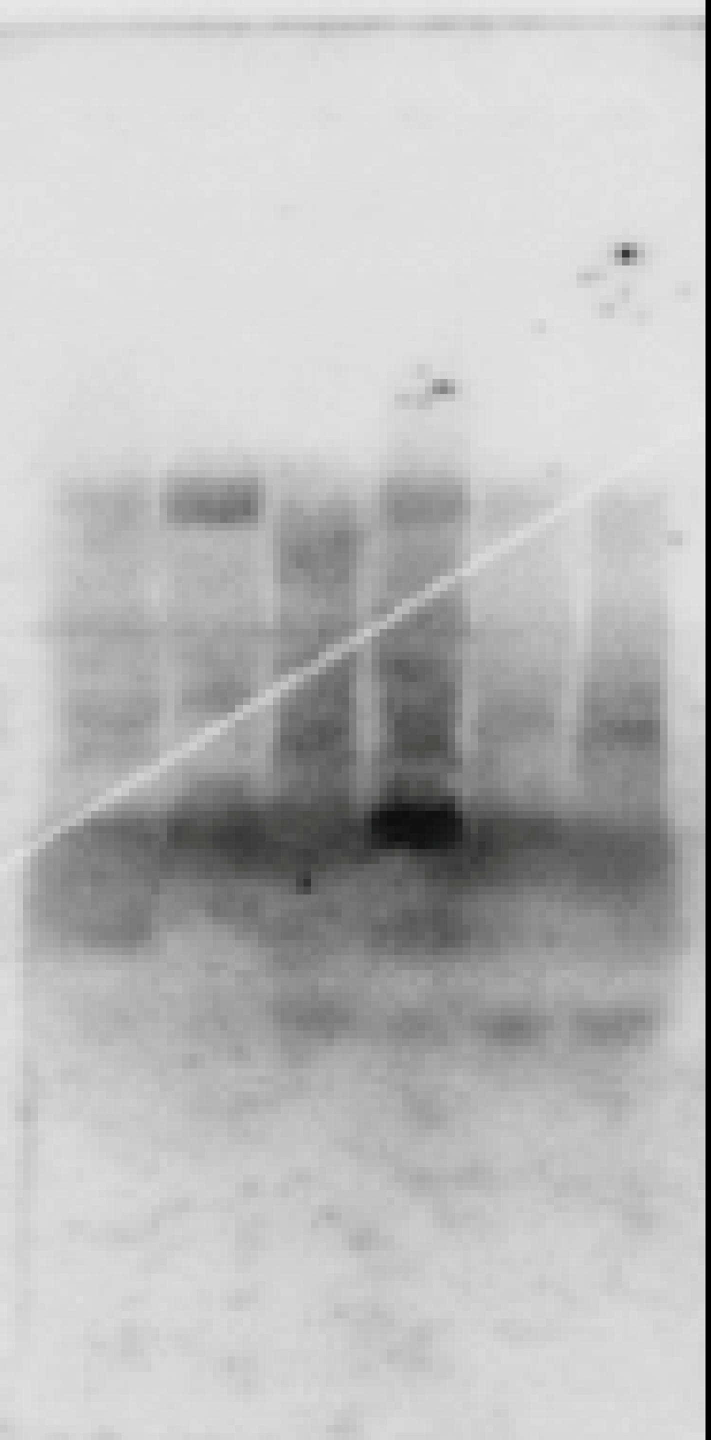

as0494

rnc 24h, 48h, 72h; wt 24h, 48h, 72h

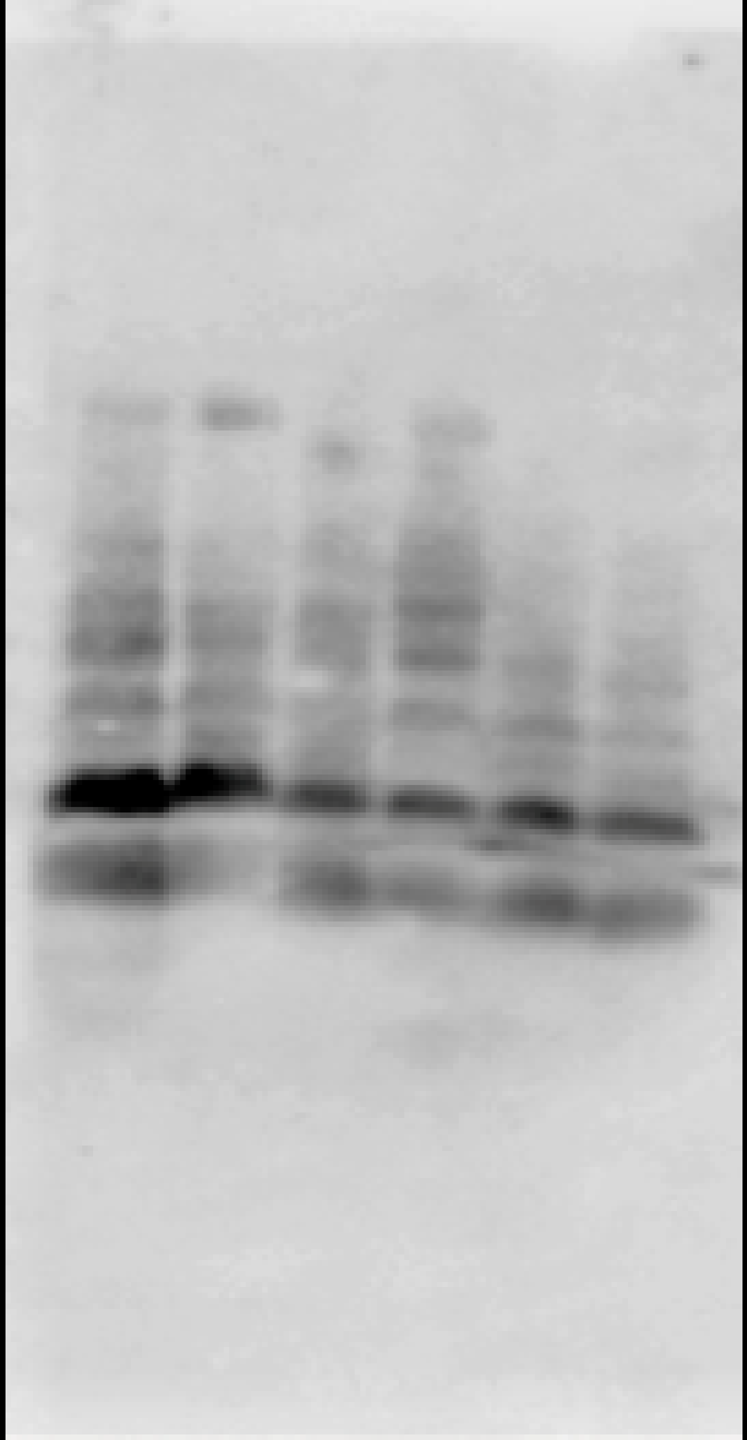

as0703

rnc 24h, 48h, 72h; wt 24h, 48h, 72h

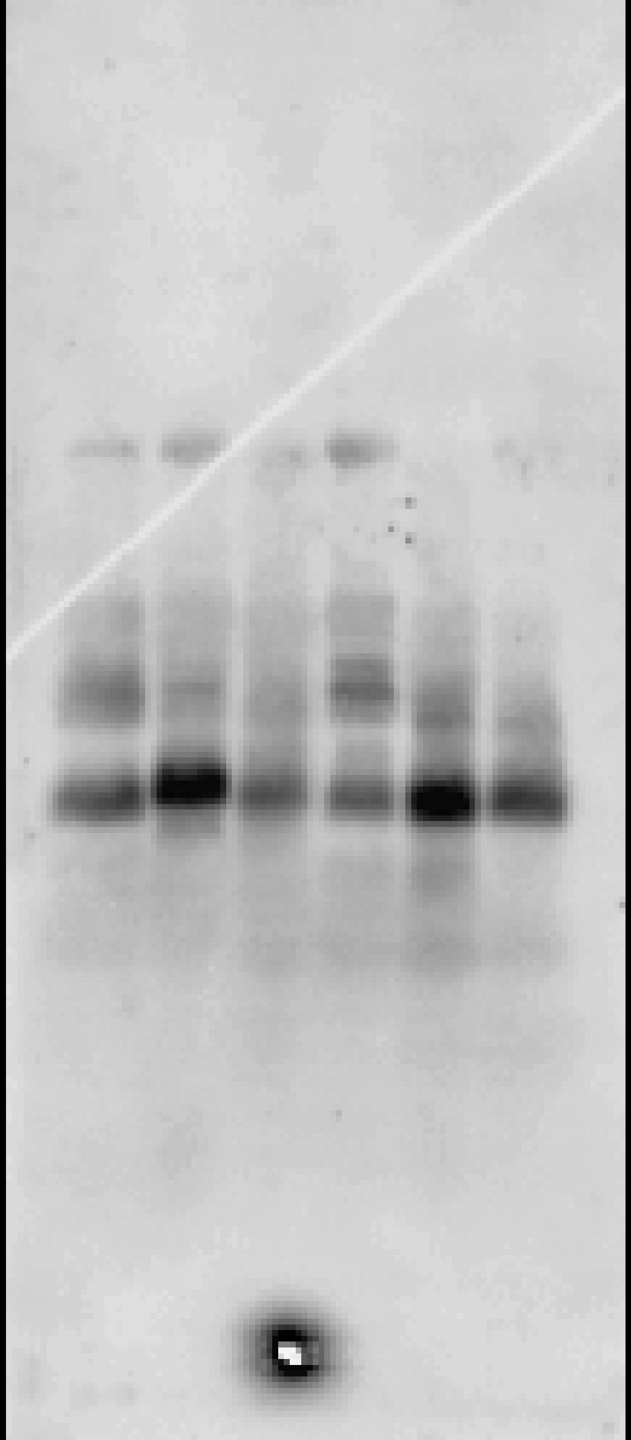

as0772

rnc 24h, 48h, 72h; wt 24h, 48h, 72h

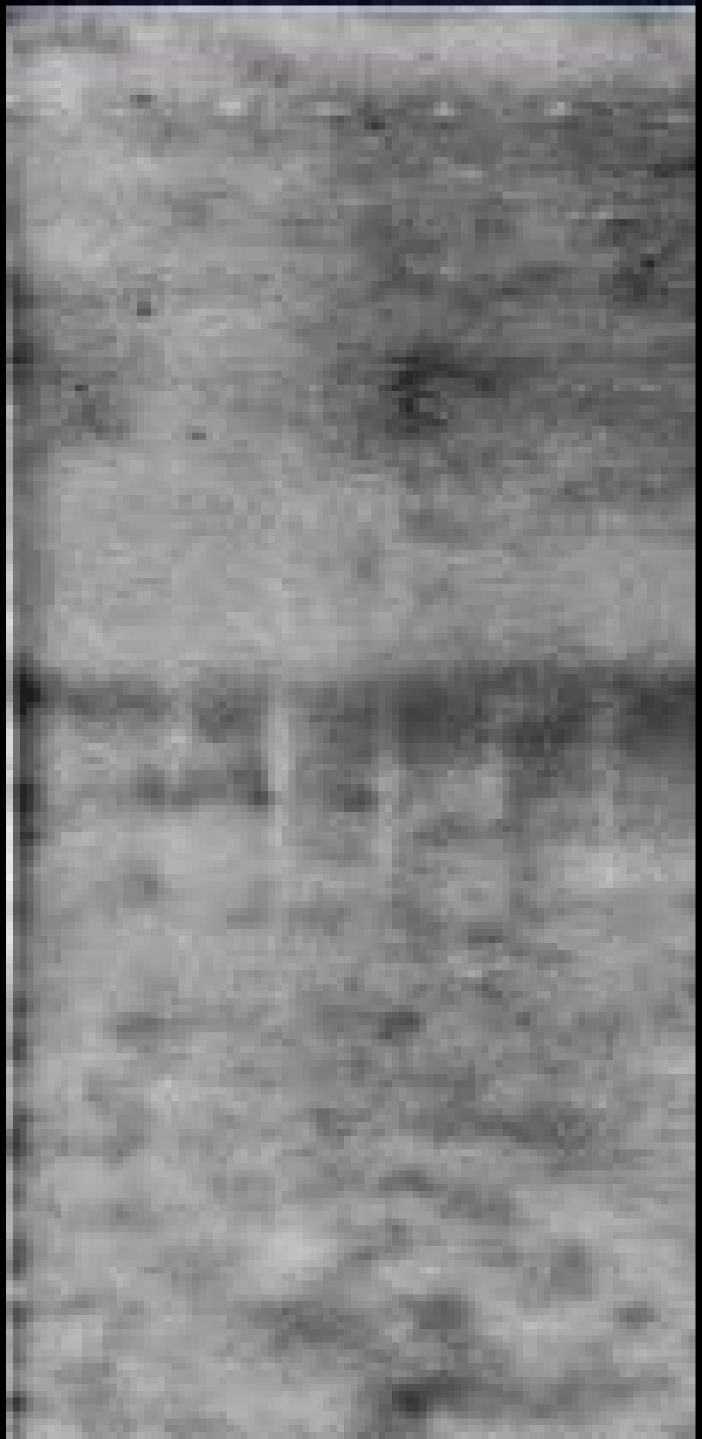

as0864

rnc 24h, 48h, 72h; wt 24h, 48h, 72h

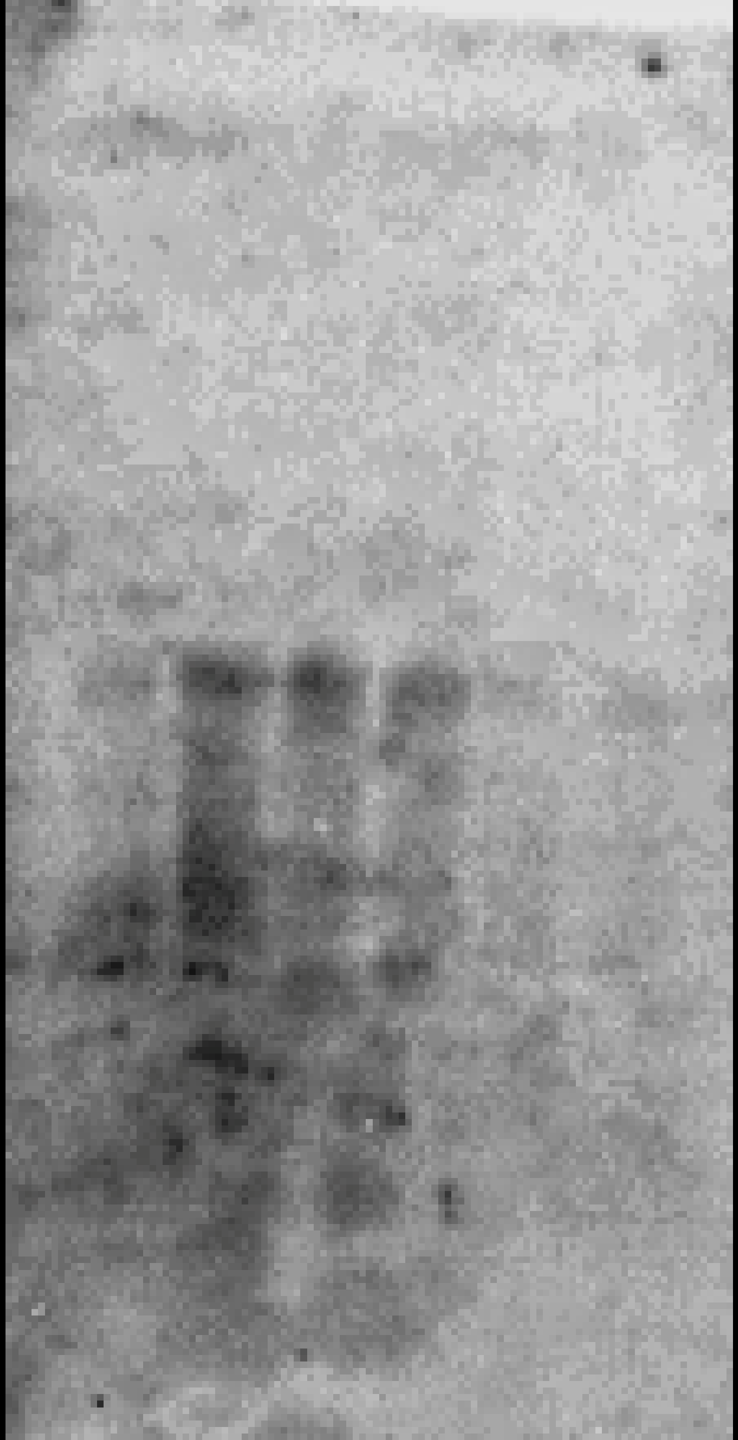

as1626

rnc 24h, 48h, 72h; wt 24h, 48h, 72h

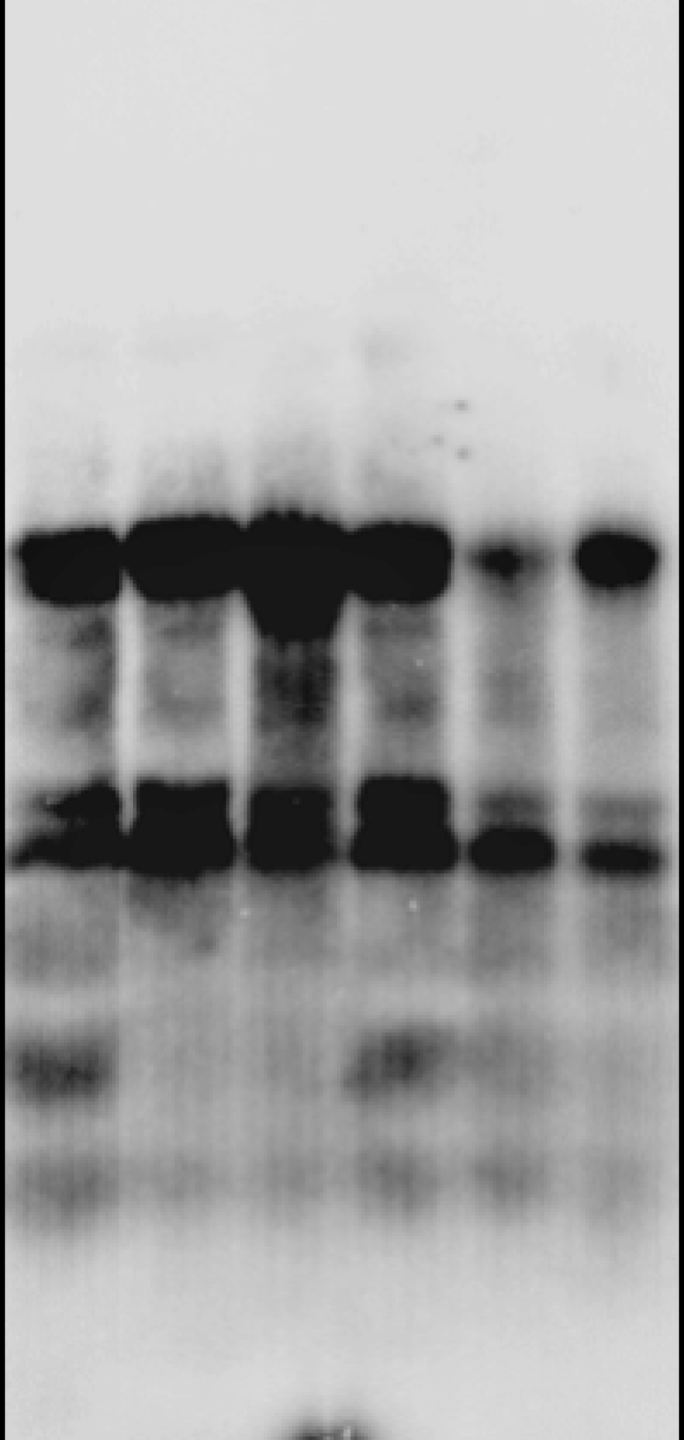

as2198

rnc 24h, 48h, 72h; wt 24h, 48h, 72h

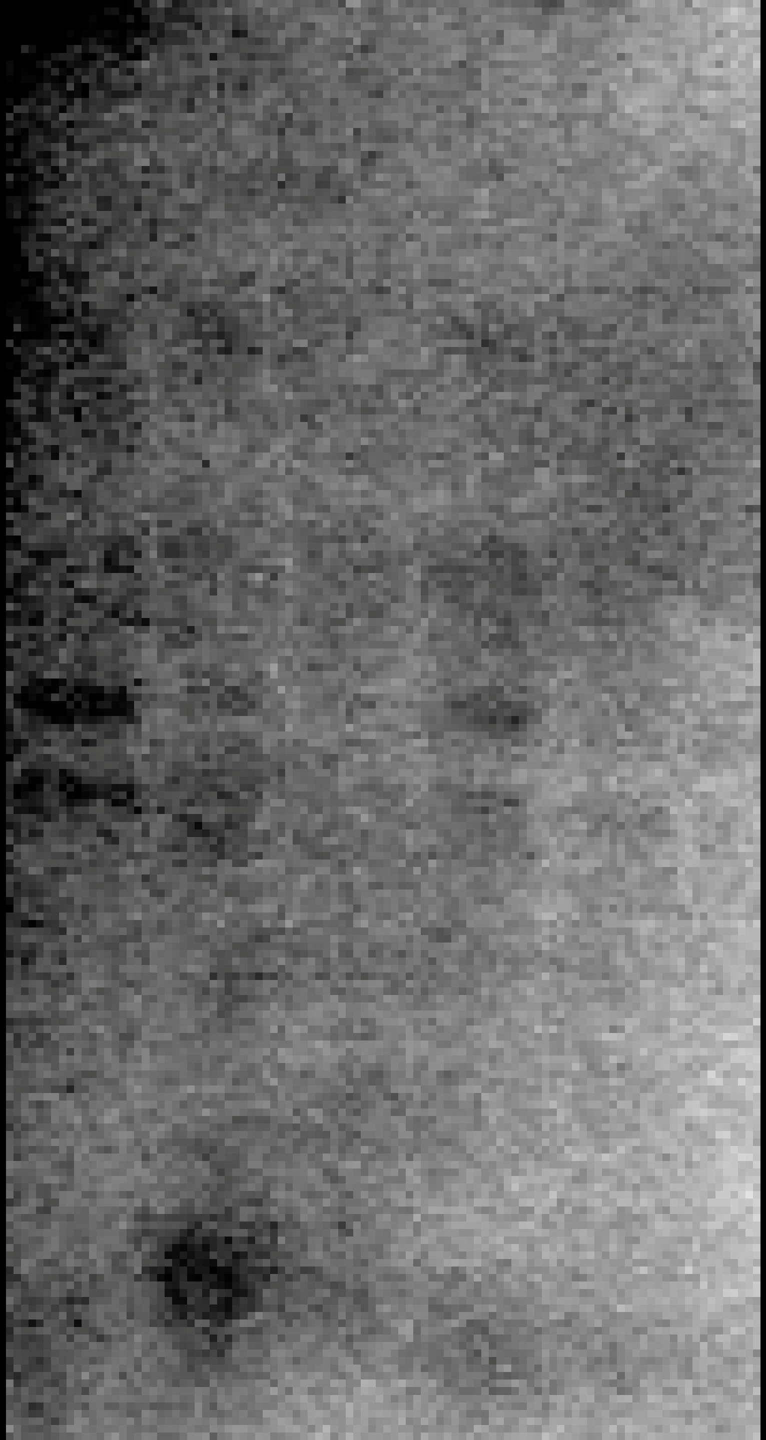

as2792

rnc 24h, 48h, 72h; wt 24h, 48h, 72h

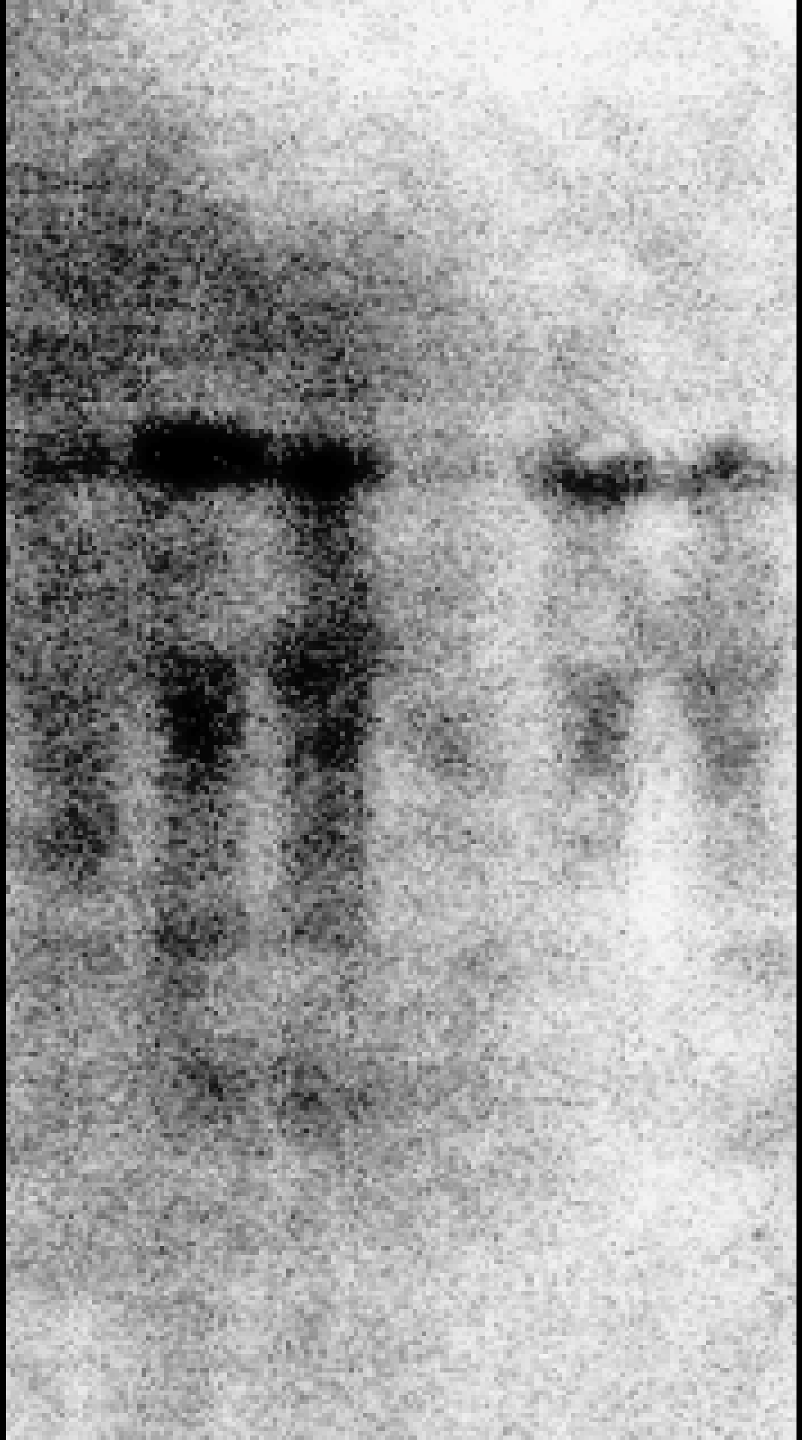

as3983

rnc 24h, 48h, 72h; wt 24h, 48h, 72h

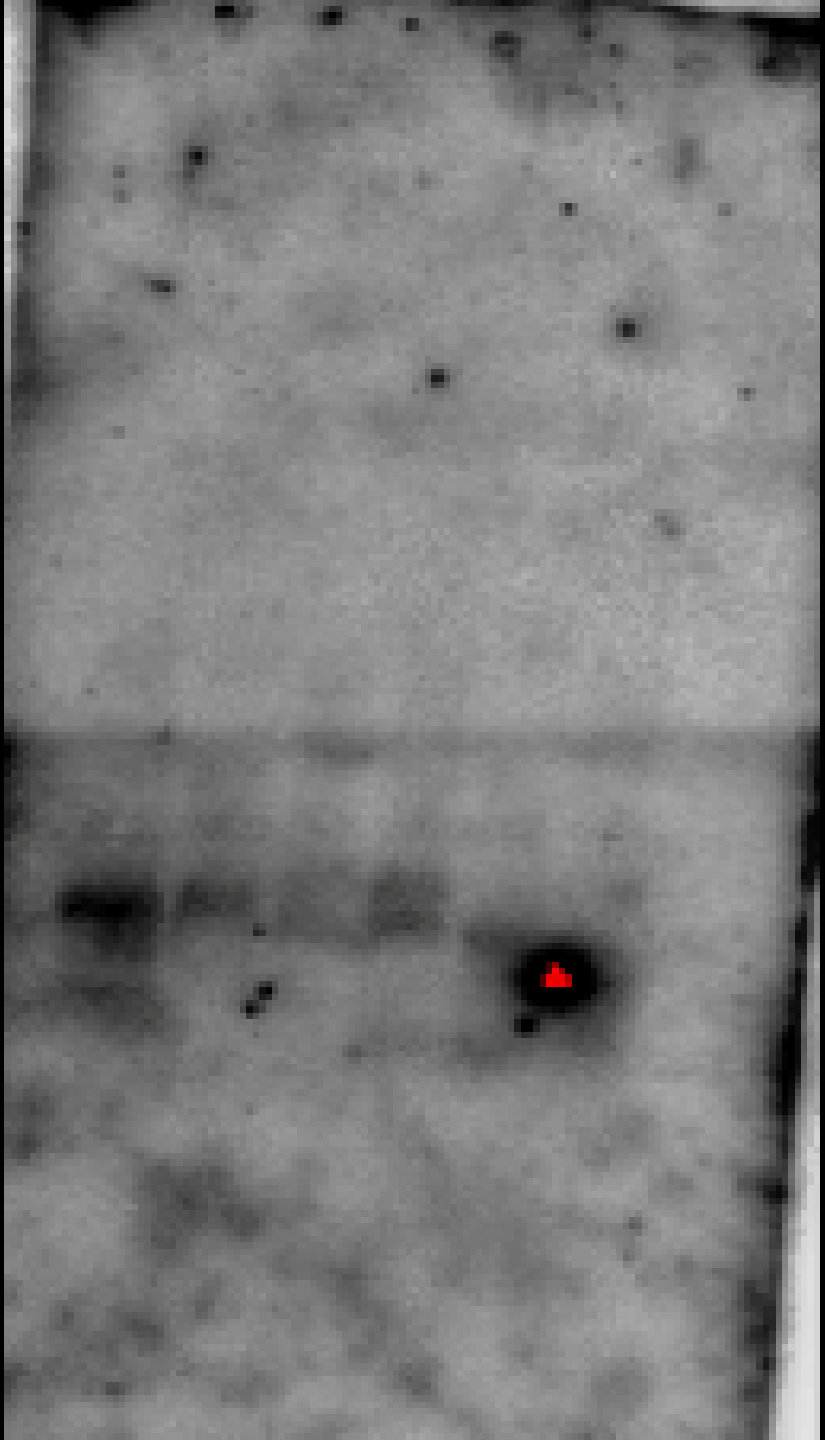

as4077

rnc 24h, 48h, 72h; wt 24h, 48h, 72h

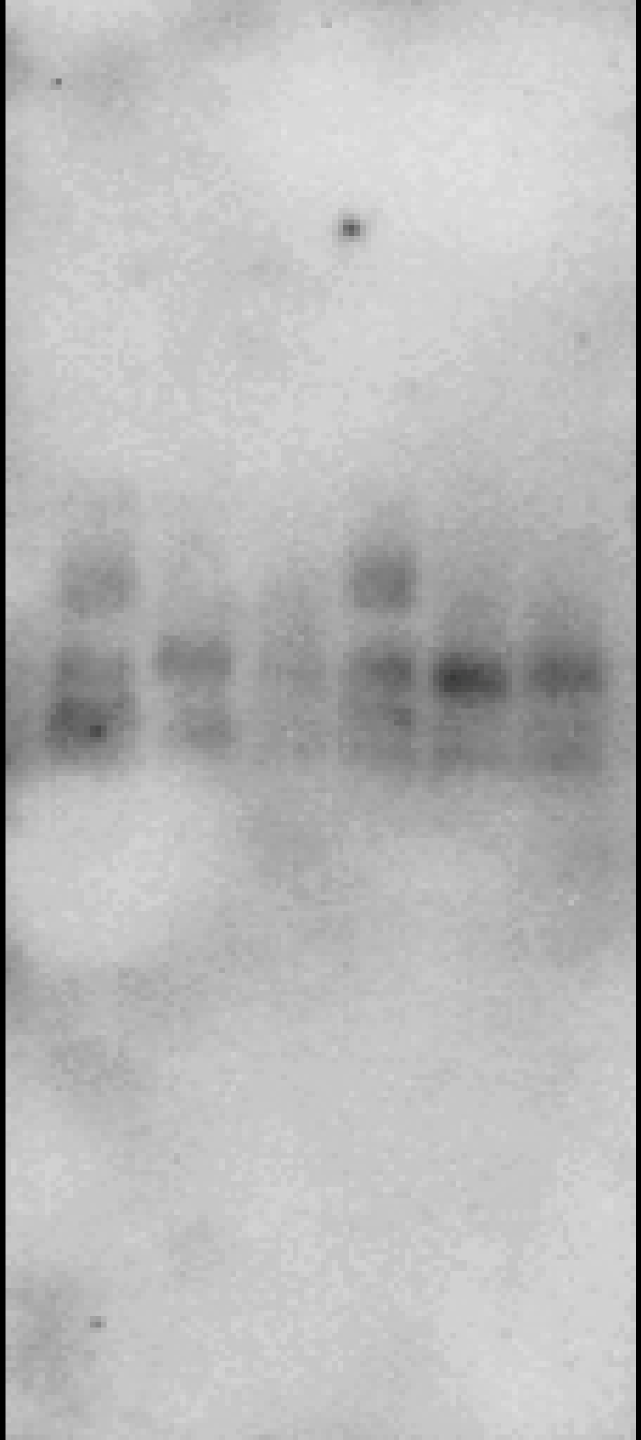

as5112

rnc 24h, 48h, 72h; wt 24h, 48h, 72h

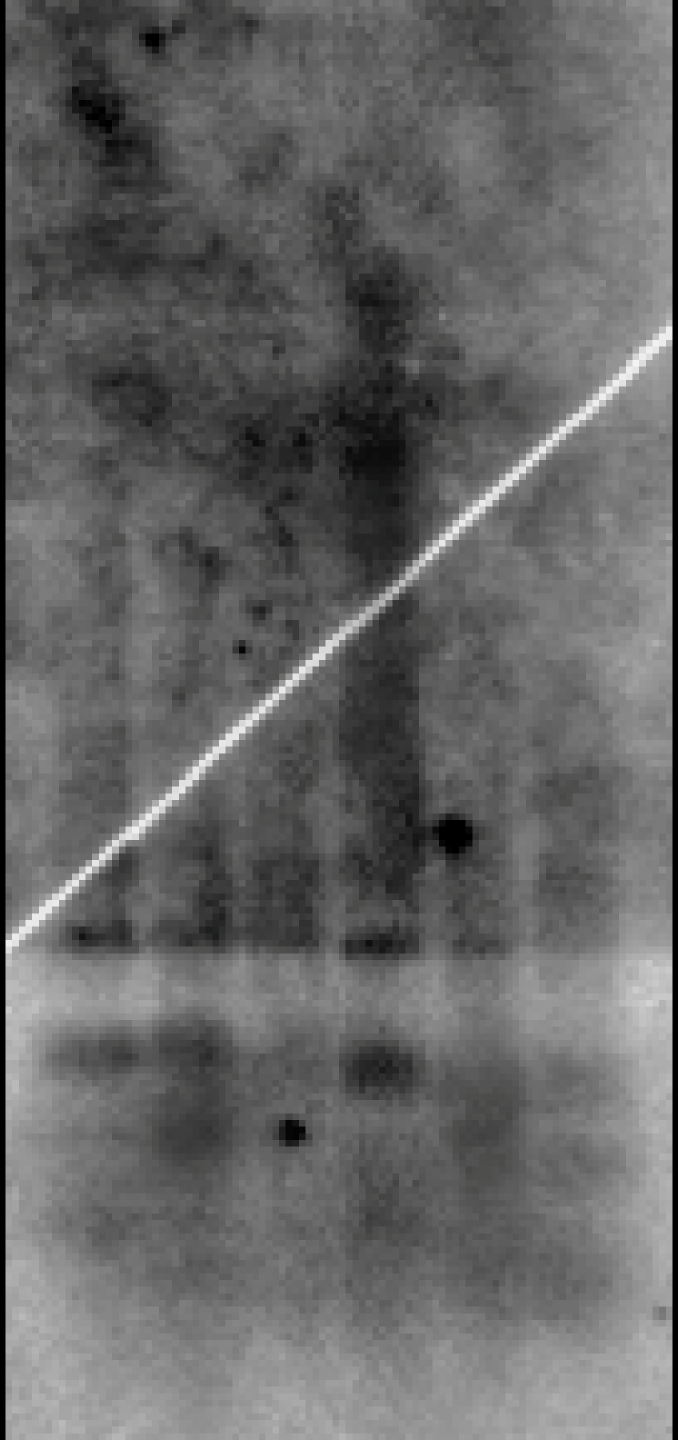

as5123

rnc 24h, 48h, 72h; wt 24h, 48h, 72h

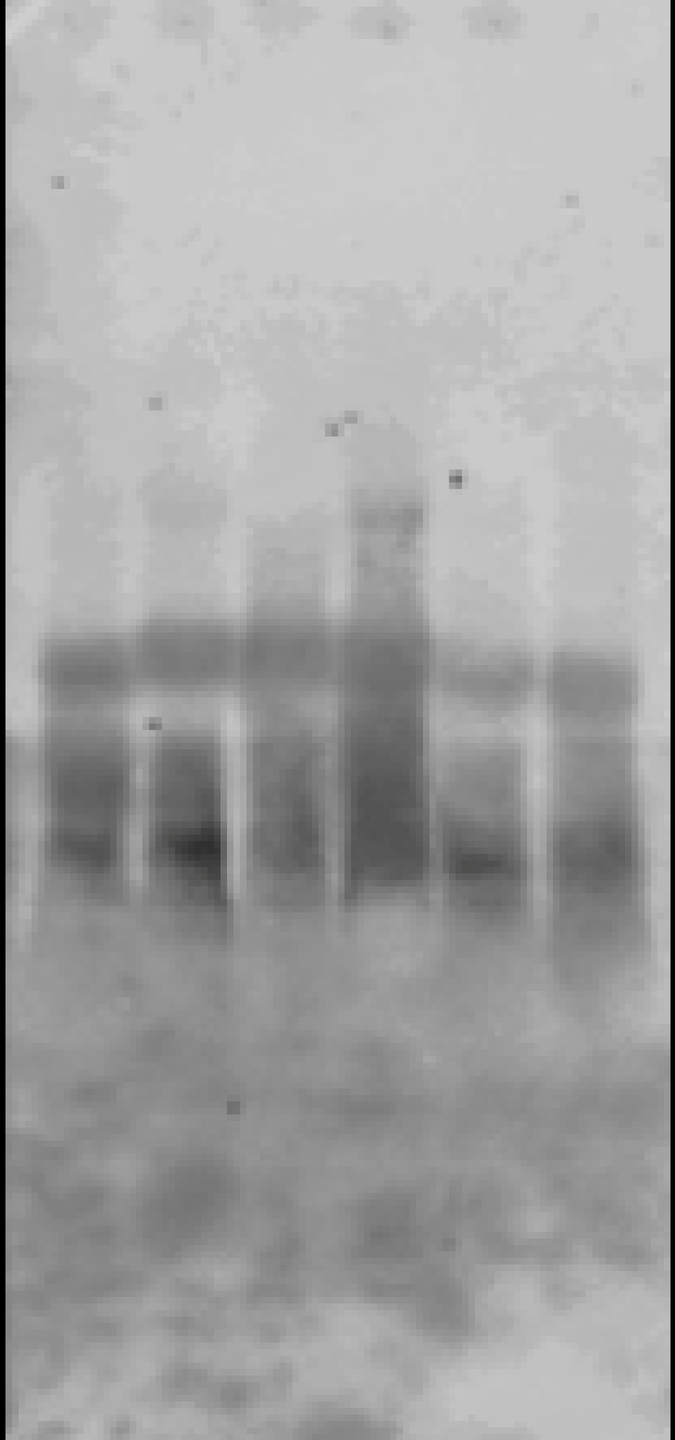

as5572

rnc 24h, 48h, 72h; wt 24h, 48h, 72h

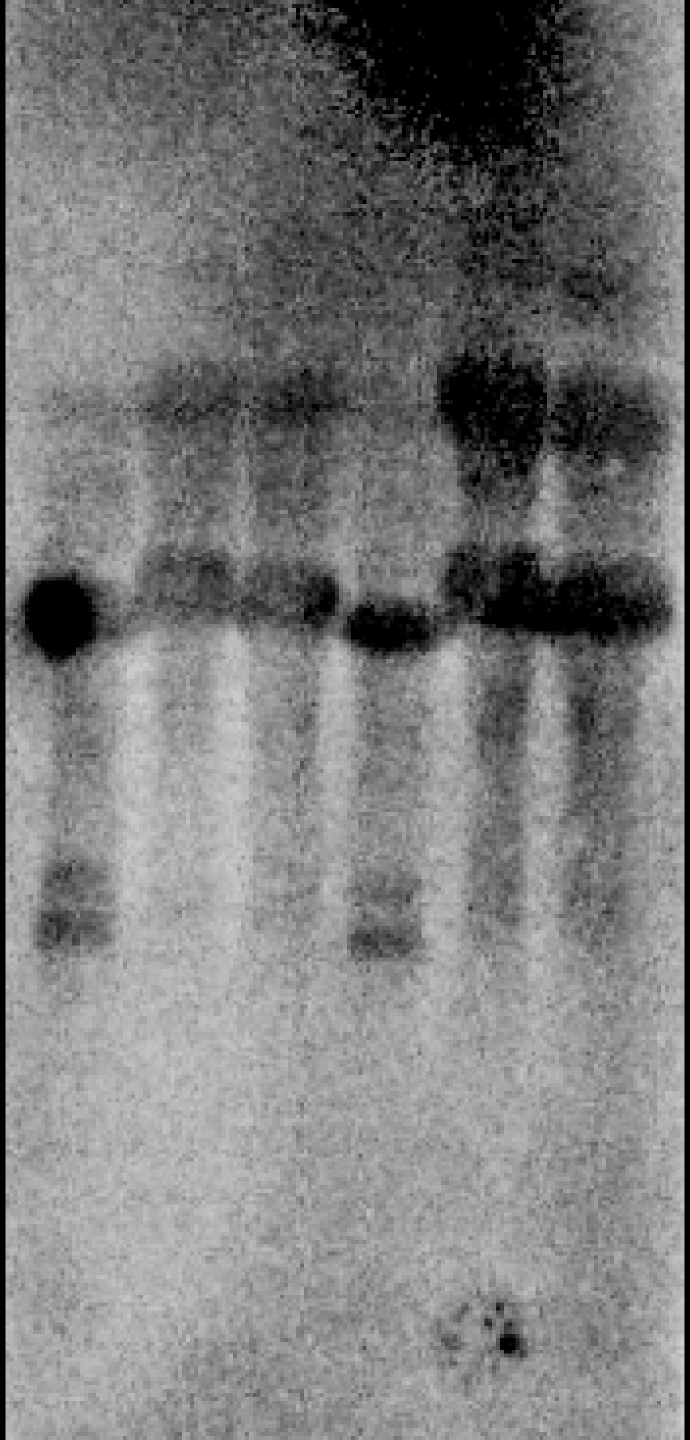

SCO0198

rnc 24h, 48h, 72h; wt 24h, 48h, 72h

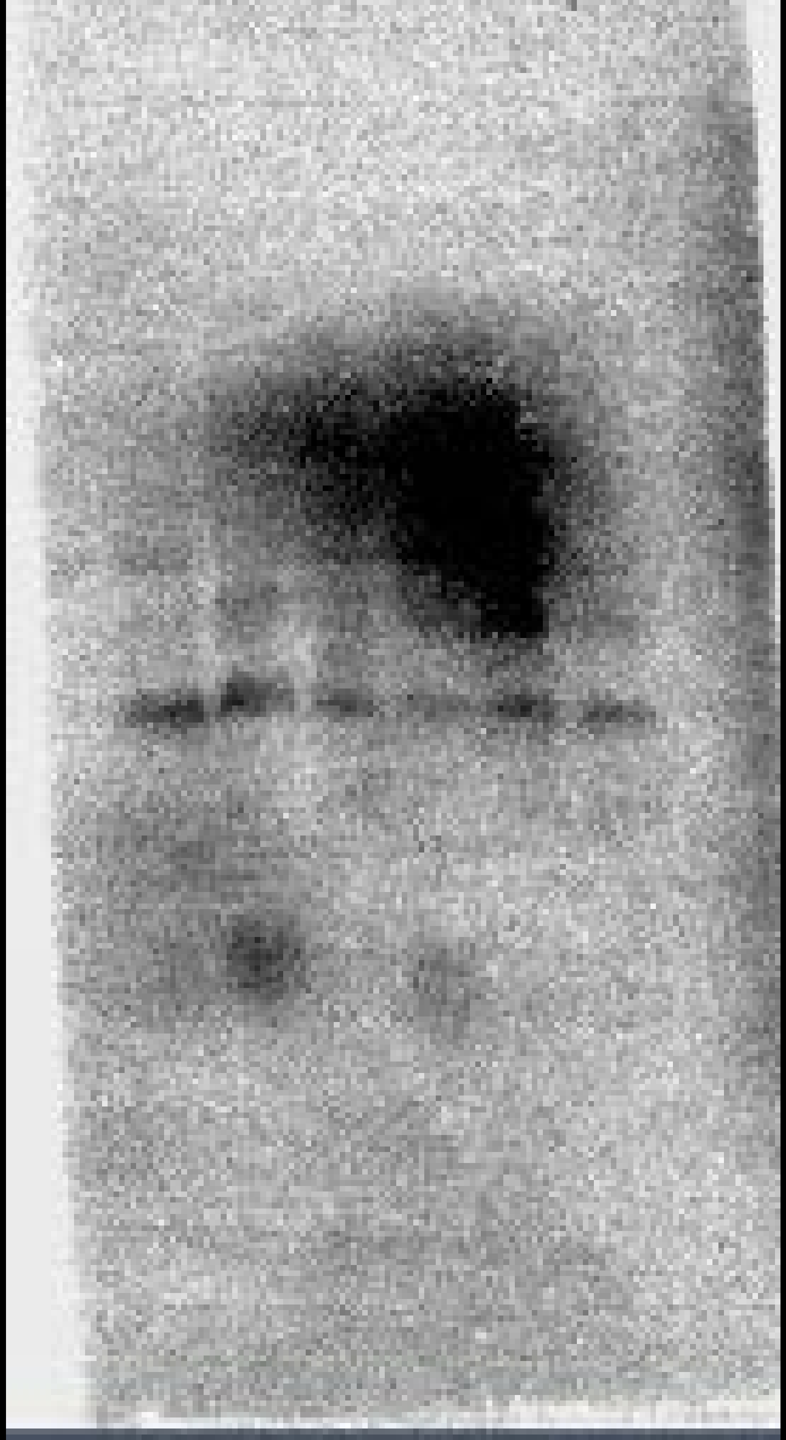

SCO0219

rnc 24h, 48h, 72h; wt 24h, 48h, 72h

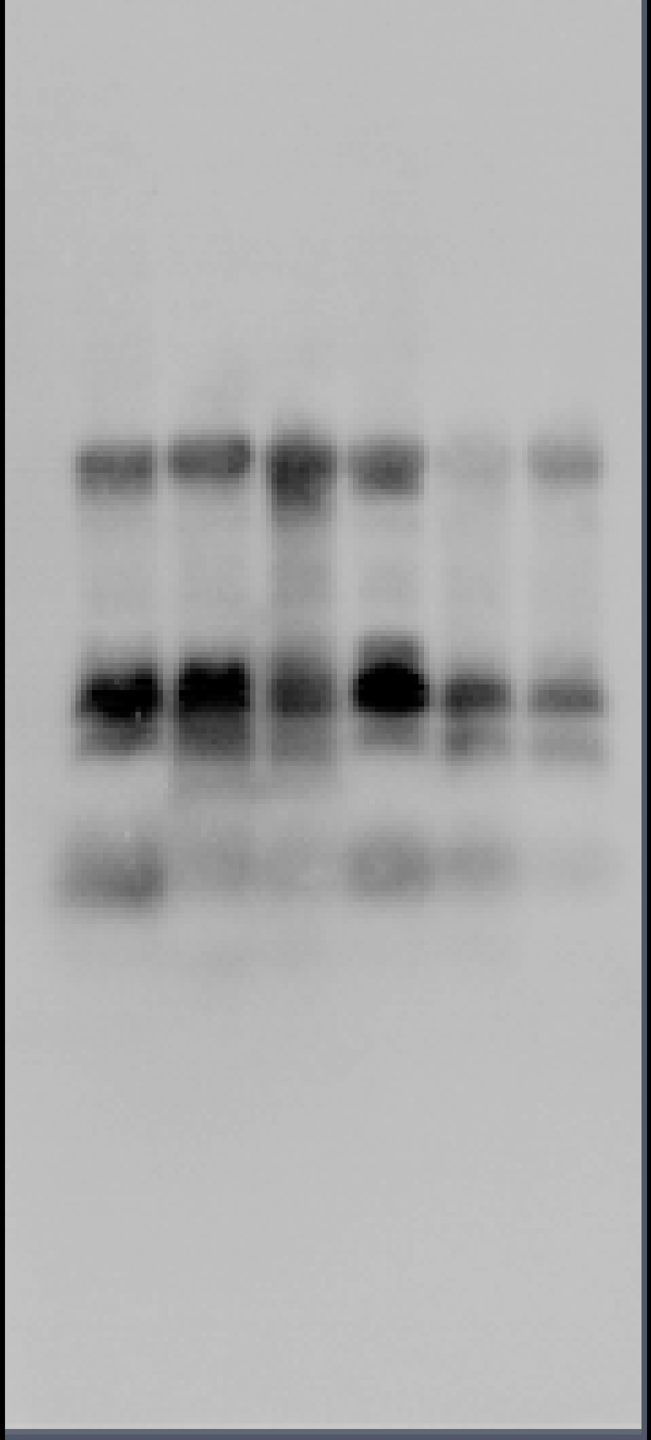

SCO0323

rnc 24h, 48h, 72h; wt 24h, 48h, 72h

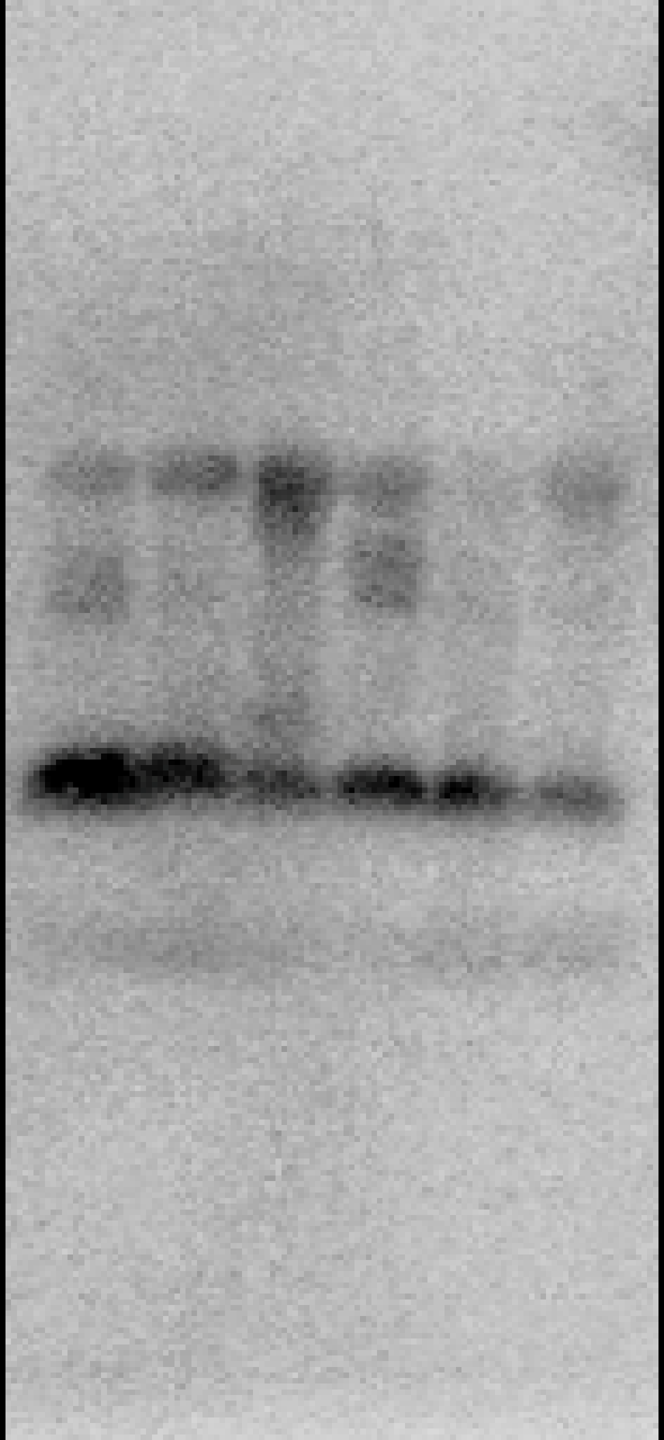

SCO0703

rnc 24h, 48h, 72h; wt 24h, 48h, 72h

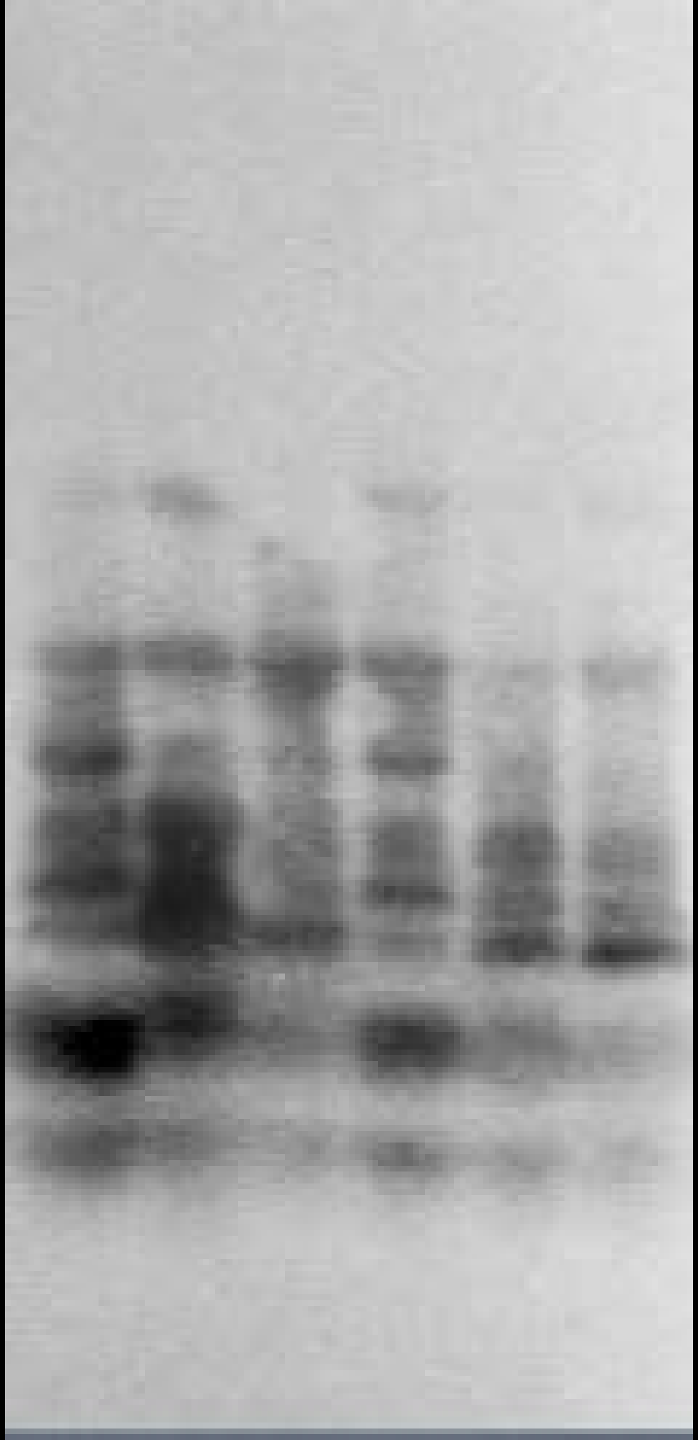

SCO0864

rnc 24h, 48h, 72h; wt 24h, 48h, 72h

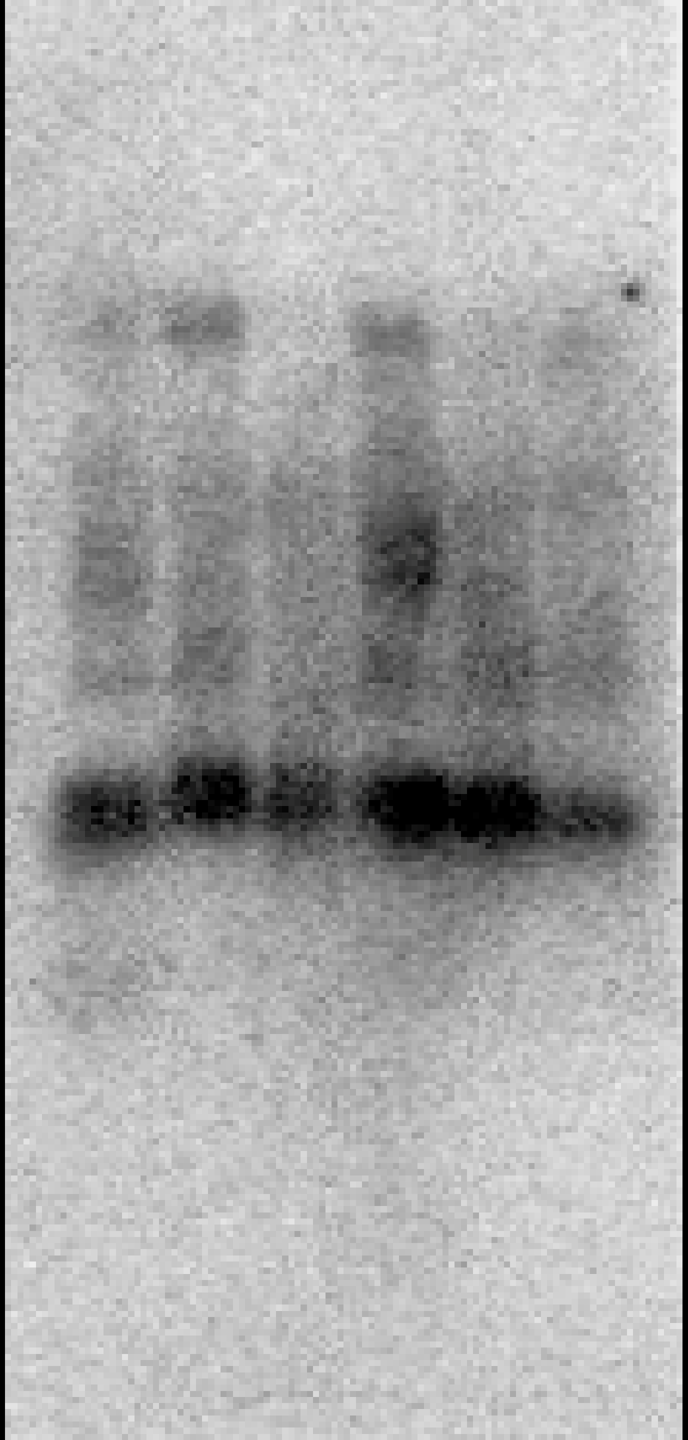

SCO2198

rnc 24h, 48h, 72h; wt 24h, 48h, 72h

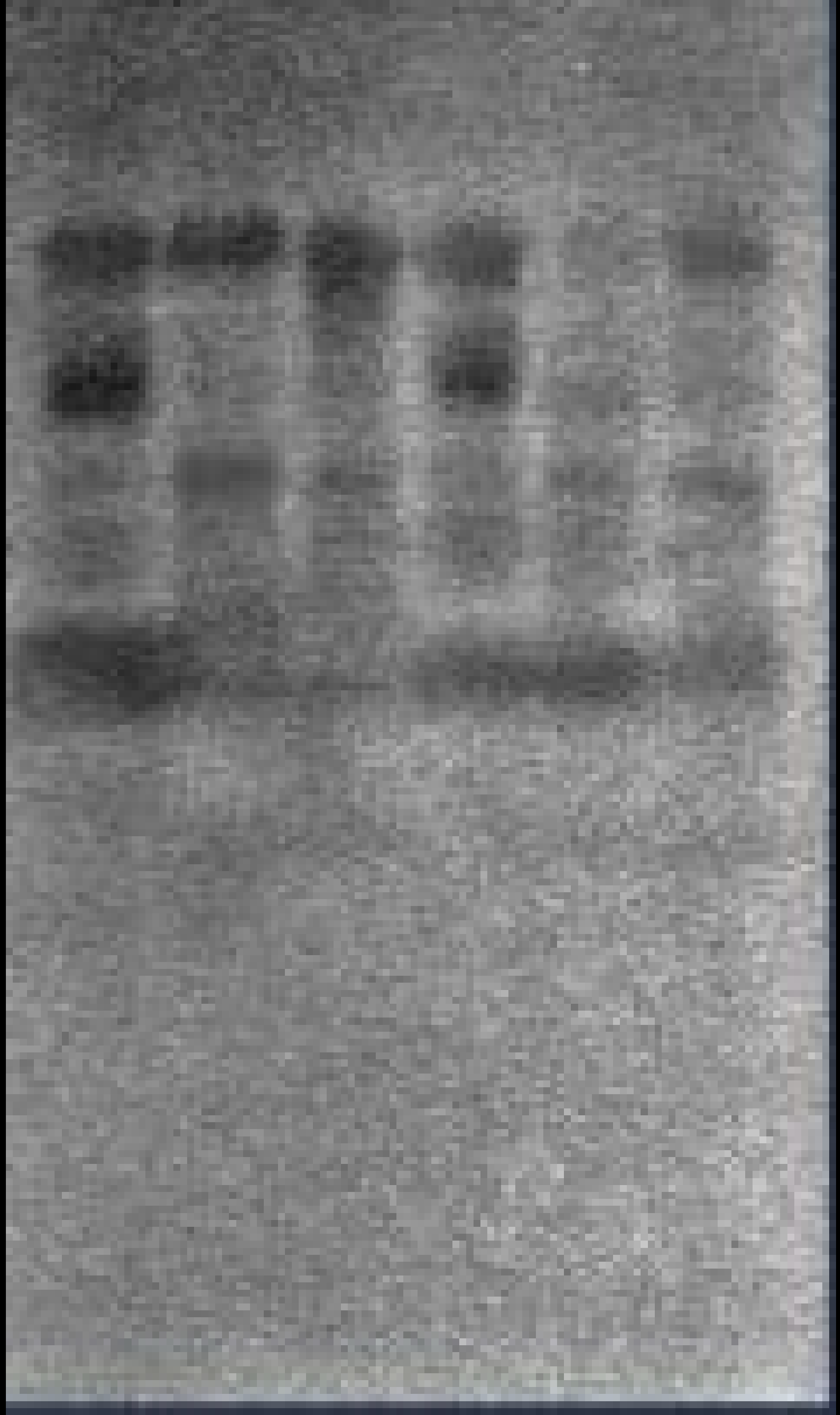

SCO2792

rnc 24h, 48h, 72h; wt 24h, 48h, 72h

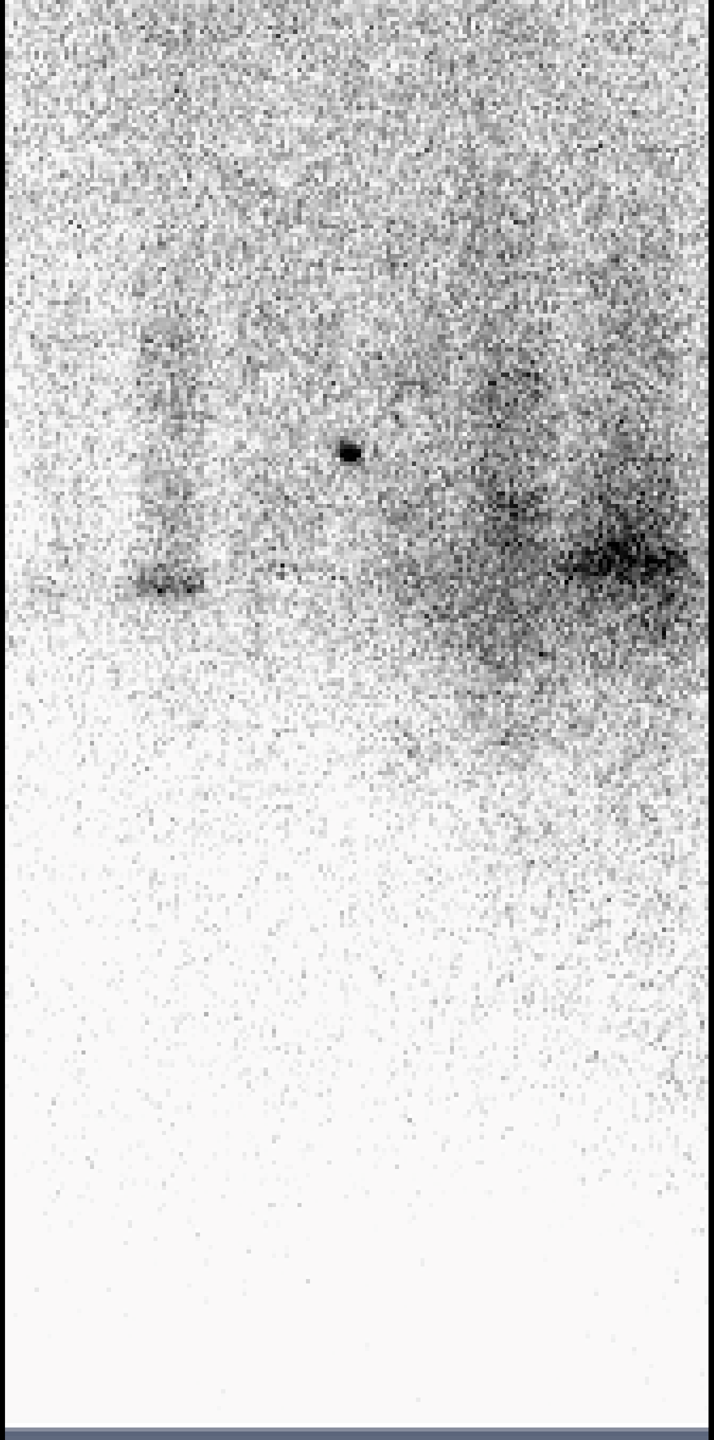

SCO3983

rnc 24h, 48h, 72h; wt 24h, 48h, 72h

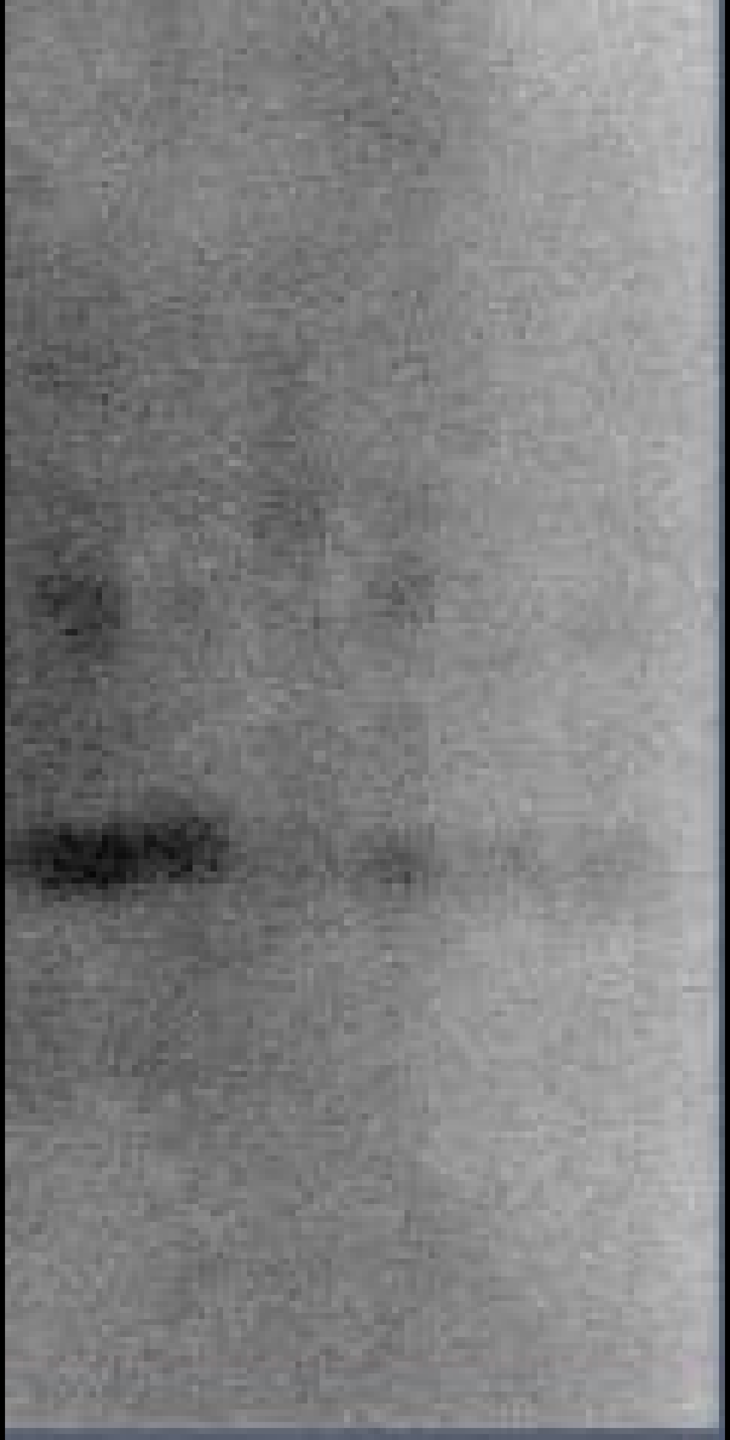

SCO4077

rnc 24h, 48h, 72h; wt 24h, 48h, 72h

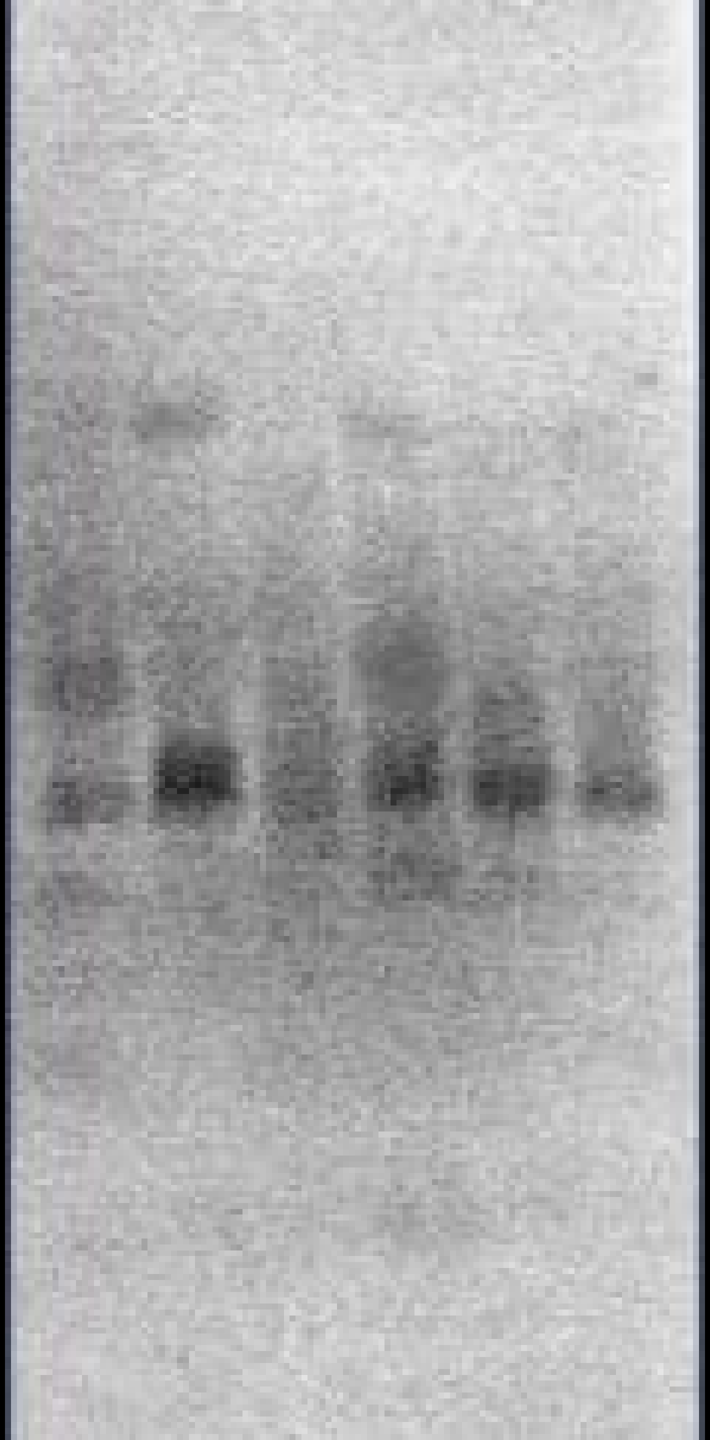

as-sigB

wt 24h, 48h, 72h; rnc 24h, 48h, 72h

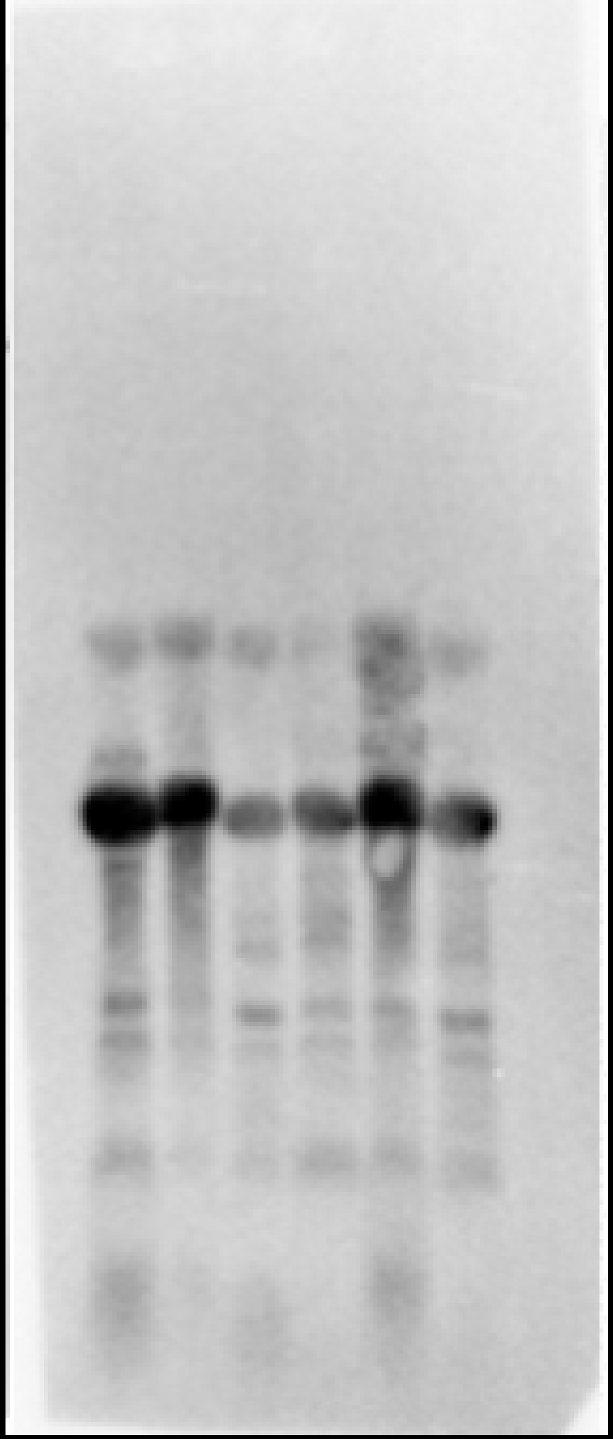

as-sigH

wt 24h, 48h, 72h; rnc 24h, 48h, 72h

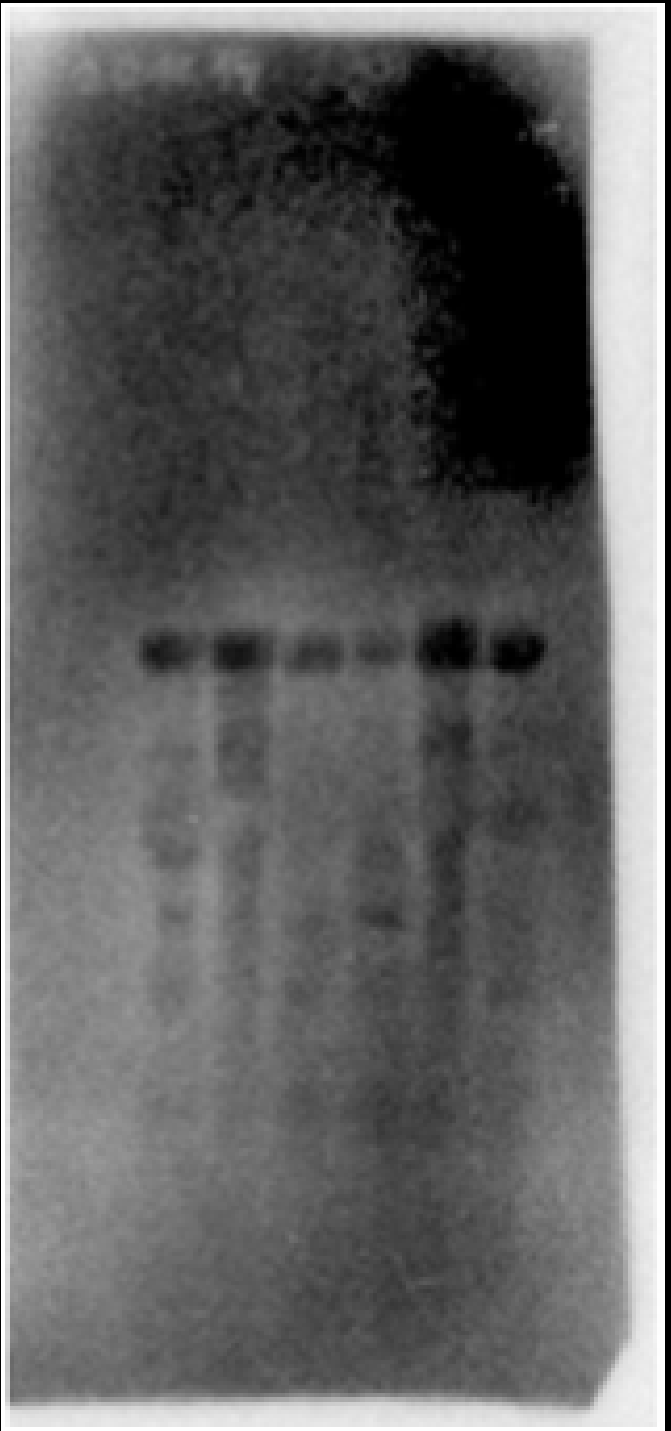

as-sigR

wt 24h, 48h, 72h; rnc 24h, 48h, 72h

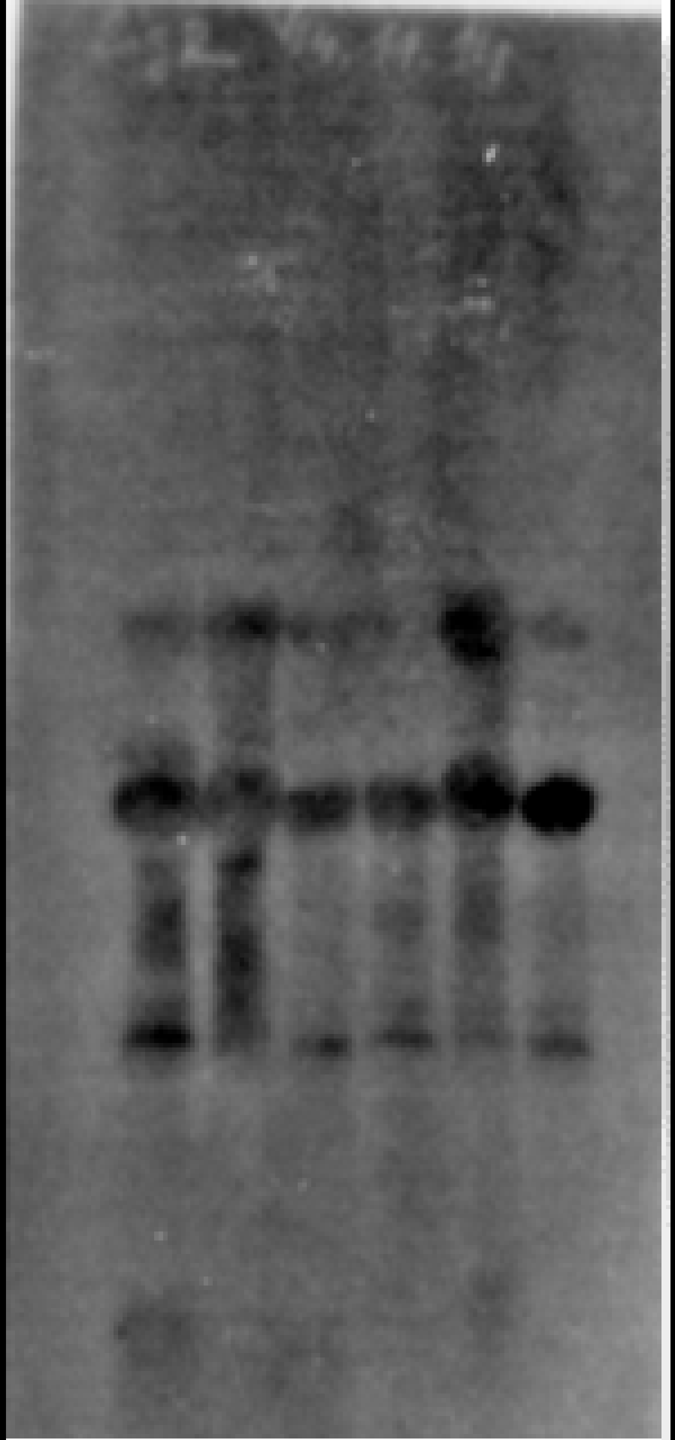

sigB

wt 24h, 48h, 72h; rnc 24h, 48h, 72h

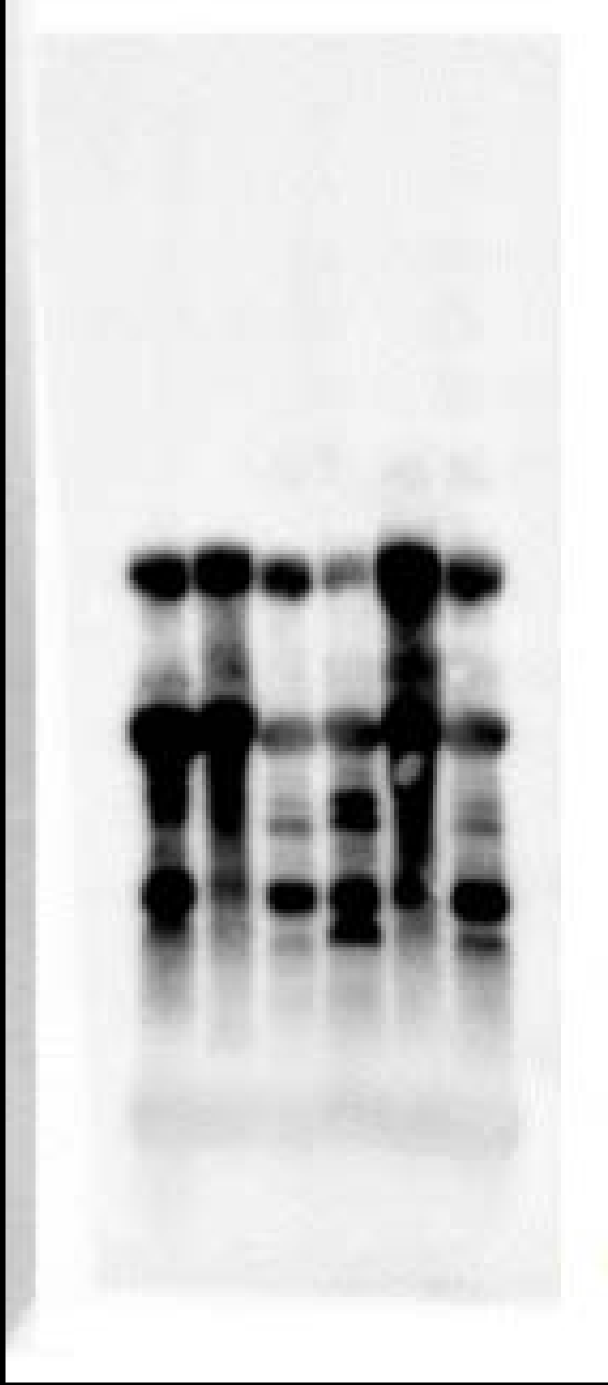

sigH

wt 24h, 48h, 72h; rnc 24h, 48h, 72h

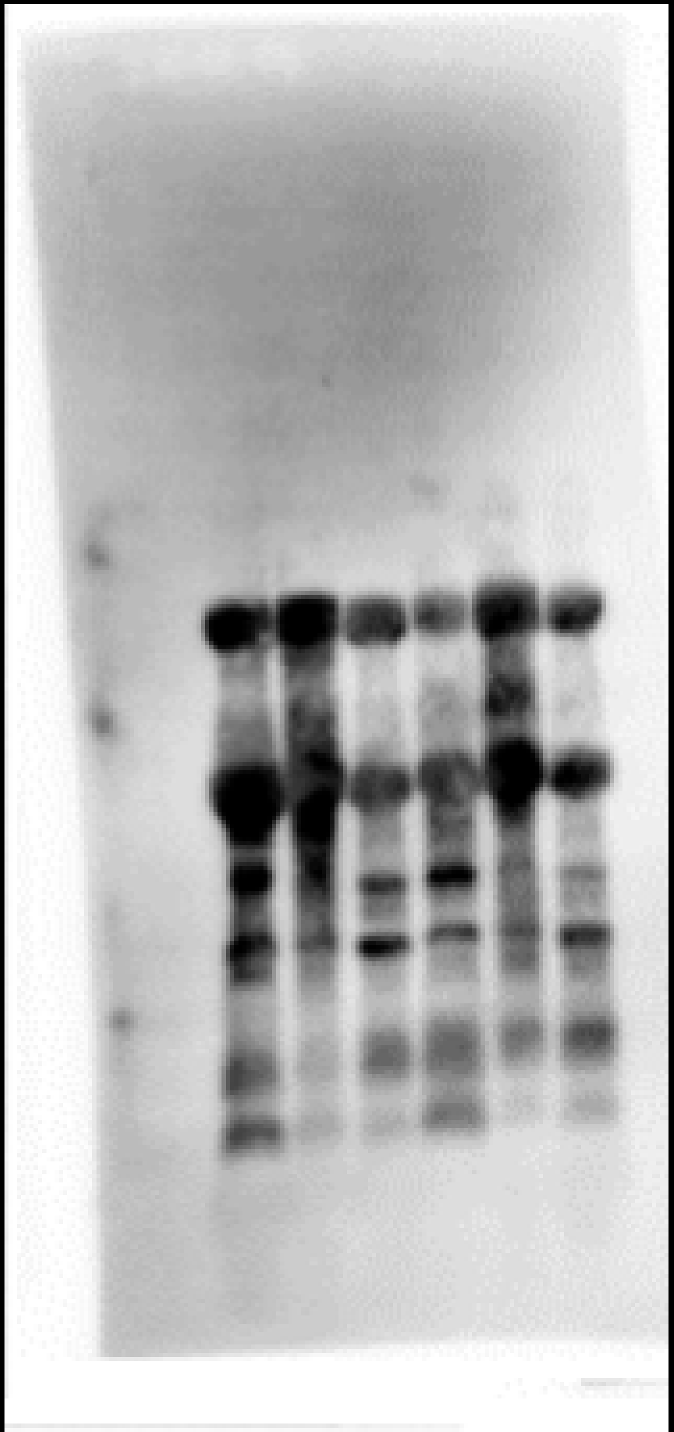

sigR

wt 24h, 48h, 72h; rnc 24h, 48h, 72h

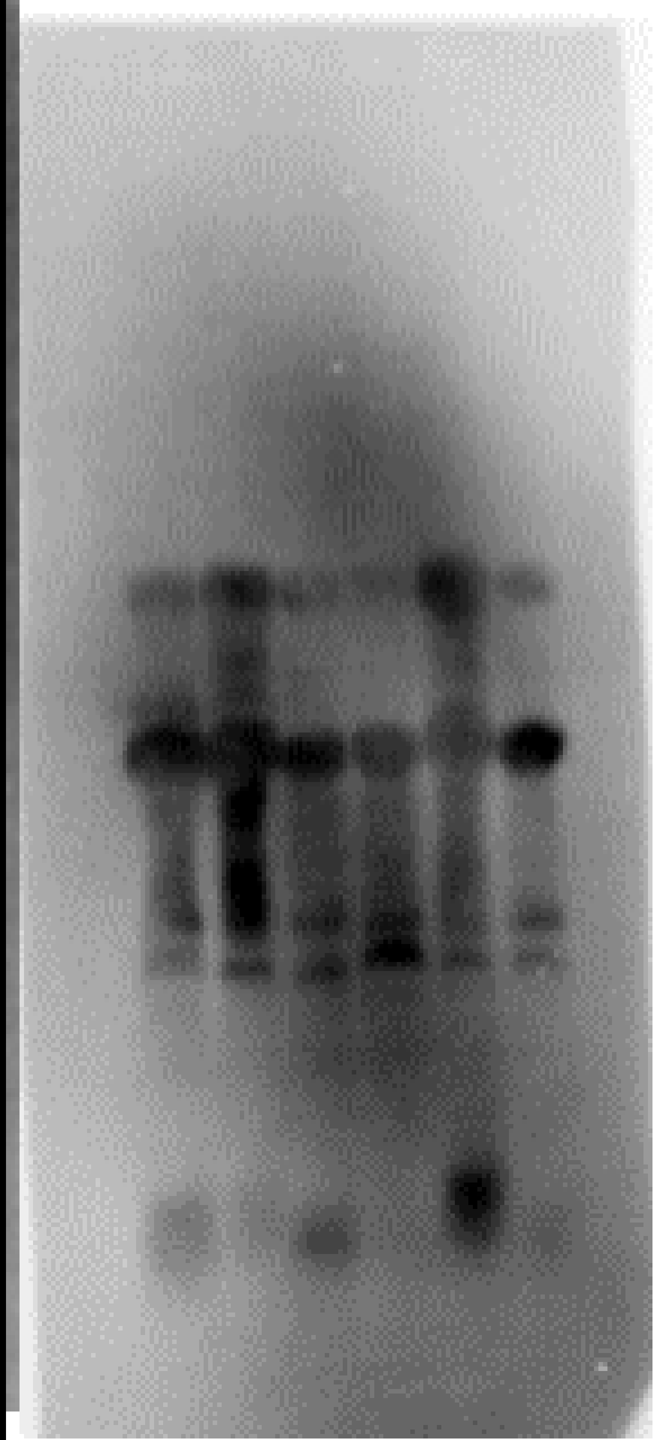

Supplement: Table S2 — Raw northern blot images. [file Presentation1.PDF]
